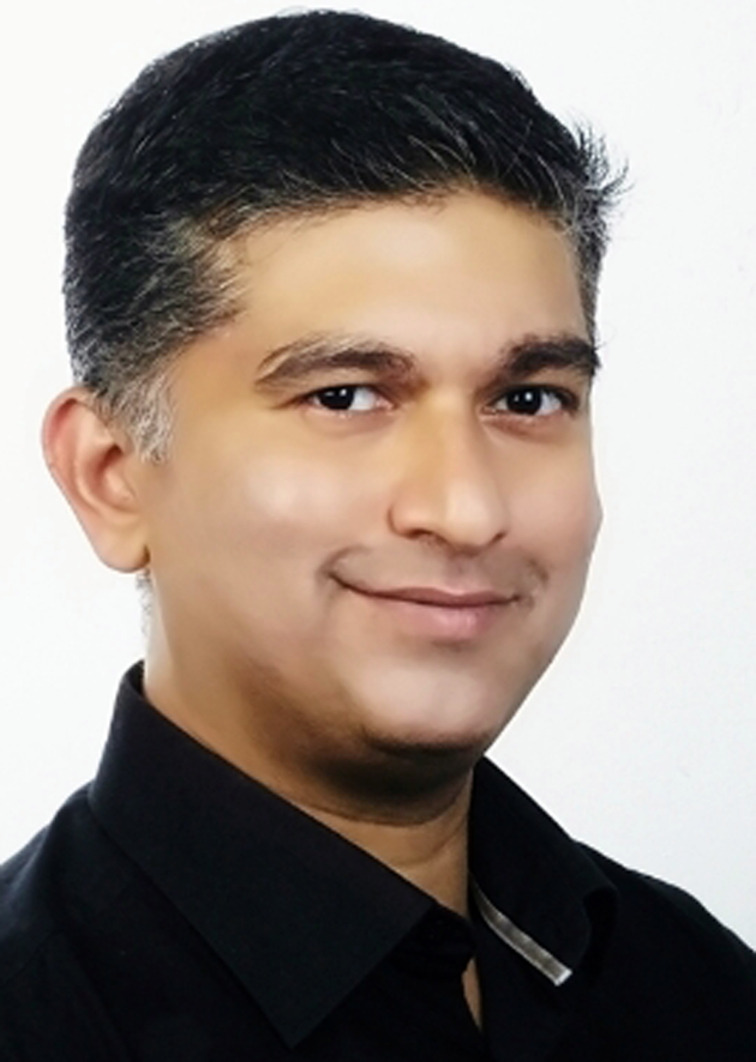# Strategies and Perspectives to Catch the Missing Pieces in Energy‐Efficient Hydrogen Evolution Reaction in Alkaline Media

**DOI:** 10.1002/anie.202015738

**Published:** 2021-02-18

**Authors:** Sengeni Anantharaj, Suguru Noda, Vasanth Rajendiran Jothi, SungChul Yi, Matthias Driess, Prashanth W. Menezes

**Affiliations:** ^1^ Department of Applied Chemistry School of Advanced Science and Engineering Waseda University 3-4-1 Okubo, Shinjuku-ku Tokyo 169-8555 Japan; ^2^ Waseda Research Institute for Science and Engineering Waseda University 3-4-1 Okubo, Shinjuku-ku Tokyo 169-8555 Japan; ^3^ Department of Chemical Engineering Hanyang University 222 Wangsimni-ro, Seongdong-gu Seoul 04763 Republic of Korea; ^4^ Department of Chemistry: Metalorganics and Inorganic Materials Technische Universität Berlin Straße des 17 Juni 135, Sekr. C2 10623 Berlin Germany

**Keywords:** electrocatalysis, heterostructured materials, hydrogen evolution reaction, transition metal hydroxides, water splitting

## Abstract

Transition metal hydroxides (M‐OH) and their heterostructures (X|M‐OH, where X can be a metal, metal oxide, metal chalcogenide, metal phosphide, etc.) have recently emerged as highly active electrocatalysts for hydrogen evolution reaction (HER) of alkaline water electrolysis. Lattice hydroxide anions in metal hydroxides are primarily responsible for observing such an enhanced HER activity in alkali that facilitate water dissociation and assist the first step, the hydrogen adsorption. Unfortunately, their poor electronic conductivity had been an issue of concern that significantly lowered its activity. Interesting advancements were made when heterostructured hydroxide materials with a metallic and or a semiconducting phase were found to overcome this pitfall. However, in the midst of recently evolving metal chalcogenide and phosphide based HER catalysts, significant developments made in the field of metal hydroxides and their heterostructures catalysed alkaline HER and their superiority have unfortunately been given negligible attention. This review, unlike others, begins with the question of why alkaline HER is difficult and will take the reader through evaluation perspectives, trends in metals hydroxides and their heterostructures catalysed HER, an understanding of how alkaline HER works on different interfaces, what must be the research directions of this field in near future, and eventually summarizes why metal hydroxides and their heterostructures are inevitable for energy‐efficient alkaline HER.

## Graphical abstract

This review brings out the key advancements made in the field of alkaline HER with metal hydroxides and their heterostructures and also provides a detailed and critical analysis of strategies and perspectives used with highlights on future prospects at the end.

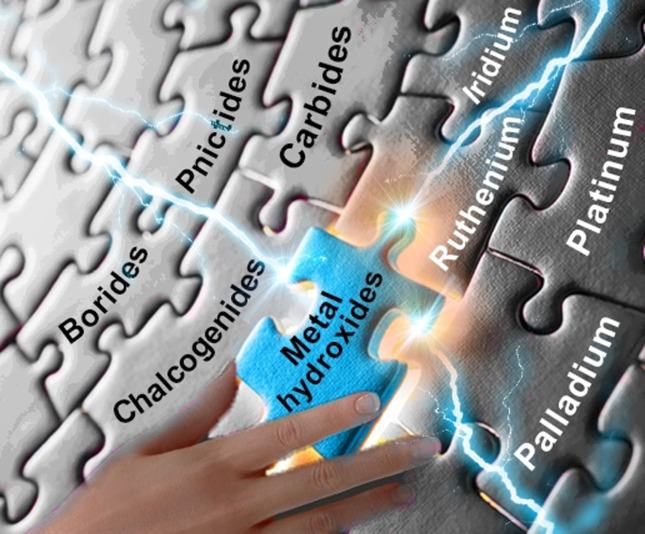

## 1 Introduction

Eco‐friendlier and efficient hydrogen production has been one of the most attention‐grabbing topics of energy research ever since the idea of hydrogen economy was coined with a vision to achieve the “affordable and clean energy” goal of 17 sustainable development goals proposed by the united nations.[^1^, ^2^] Current cost‐efficient method, the steam reforming of methane provides affordable hydrogen at the cost of low purity.[^3^, ^4^, ^5^, ^6^] Besides, it also requires methane/other low molecular weight hydrocarbons that are quickly depleting or more precisely being overconsumed with an exponentially increasing rate. This suggests that steam reforming thereby cannot be a viable option for all our future hydrogen demand.[^7^, ^8^] A similar method that primarily consists of steam reforming for hydrogen production is biomass gasification and catalytic reforming under steam.[^9^, ^10^] Though this method seems to be a practical option as biomass production is anticipated to increase linearly with the increasing population, the amount of hydrogen that can be produced by this method will never be sufficient to meet the demand in the future. Biomass gasification is carbon‐neutral, however, both steam reforming and biomass gasification release greenhouse gases (GHGs) and hence, they are not environmentally friendlier.[^11^, ^12^] Because of the aforementioned disadvantages albeit having cost‐efficiencies, steam reforming of methane and biomass gasification are to be replaced with an advantageous method that produces hydrogen with the highest purity and emits no GHGs. A method that fits within these criteria is electrocatalytic water splitting.[^13^, ^14^, ^15^, ^16^, ^17^, ^18^, ^19^]

Electrocatalytic water splitting is regarded as the fastest, safest, and the greenest (provided that input energy is from renewables) method of all capable of producing highly pure hydrogen (99.999 %) while also being regarded as an indirect mean of large‐scale energy storage that stores electrical energy as chemical fuels.[^20^, ^21^] In spite of having such advantages, water electrosplitting lacks in energy‐efficiency and also suffers from a very low abundance of the best active catalysts which have been the main reasons for hindering its successful commercialization.[^22^, ^23^, ^24^] Affordability of hydrogen produced during catalytic water electrosplitting is determined primarily by the activity and the availability of electrode materials used.[^25^, ^26^] Materials that perform the half‐cell reactions (oxygen evolution reaction (OER) at the anode and hydrogen evolution reaction (HER) at the cathode) with a better energy efficiency (i.e., with lower overpotentials) involve Pt (for HER) and the oxides of Ir and Ru (for OER). These metals are very low in abundance that forbids the successful scale‐up of water electrolysers for large‐scale hydrogen production.[^25^, ^26^] To avoid this issue with these precious metals based electrocatalysts, several strategies were proposed and noteworthy ones are nanostructuring and noble metal dilution by alloying and soft‐templating in colloidal solutions.[^27^, ^28^, ^29^, ^30^, ^31^] Energy‐efficiency of hydrogen production is also improved by performing the electrolysis with aqueous solutions of extremely low or high pH (such as 0 (e.g. 0.5 M H_2_SO_4_ or 1.0 M HClO_4_) and 14 (e.g. 1.0 M KOH)) and the recent developments in the area of proton exchange membrane (PEM) for acidic water electrolysis and alkaline water electrolysis equipped with anion exchange membranes (AEM) are significant in the past two decades.[^20^, ^32^, ^33^] Besides, a remarkable amount of research works is also being done in the area of neutral and near‐neutral electrocatalytic water splitting with a vision of improving the efficiency of solar to fuel conversion (SFC) devices, the activity of which is largely dependent on the electrocatalytic activity of materials used in neutral and near‐neutral waters.[^13^, ^34^, ^35^, ^36^, ^37^, ^38^]

Acidic water electrolysis is blessed with an extensive library of recently evolved non‐precious HER catalysts that perform better than Pt in acid.[^7^, ^8^] However, it still lacks in the development of highly active and reasonably stable OER catalysts made of cheaper materials. Known acidic OER catalysts of cheaper materials often contain the oxides of Co and Mn with other post‐transition elements that fetch the ability to self‐heal under a highly corrosive acidic environment under oxidizing potentials.[^39^, ^40^, ^41^]

**Scheme 1:**
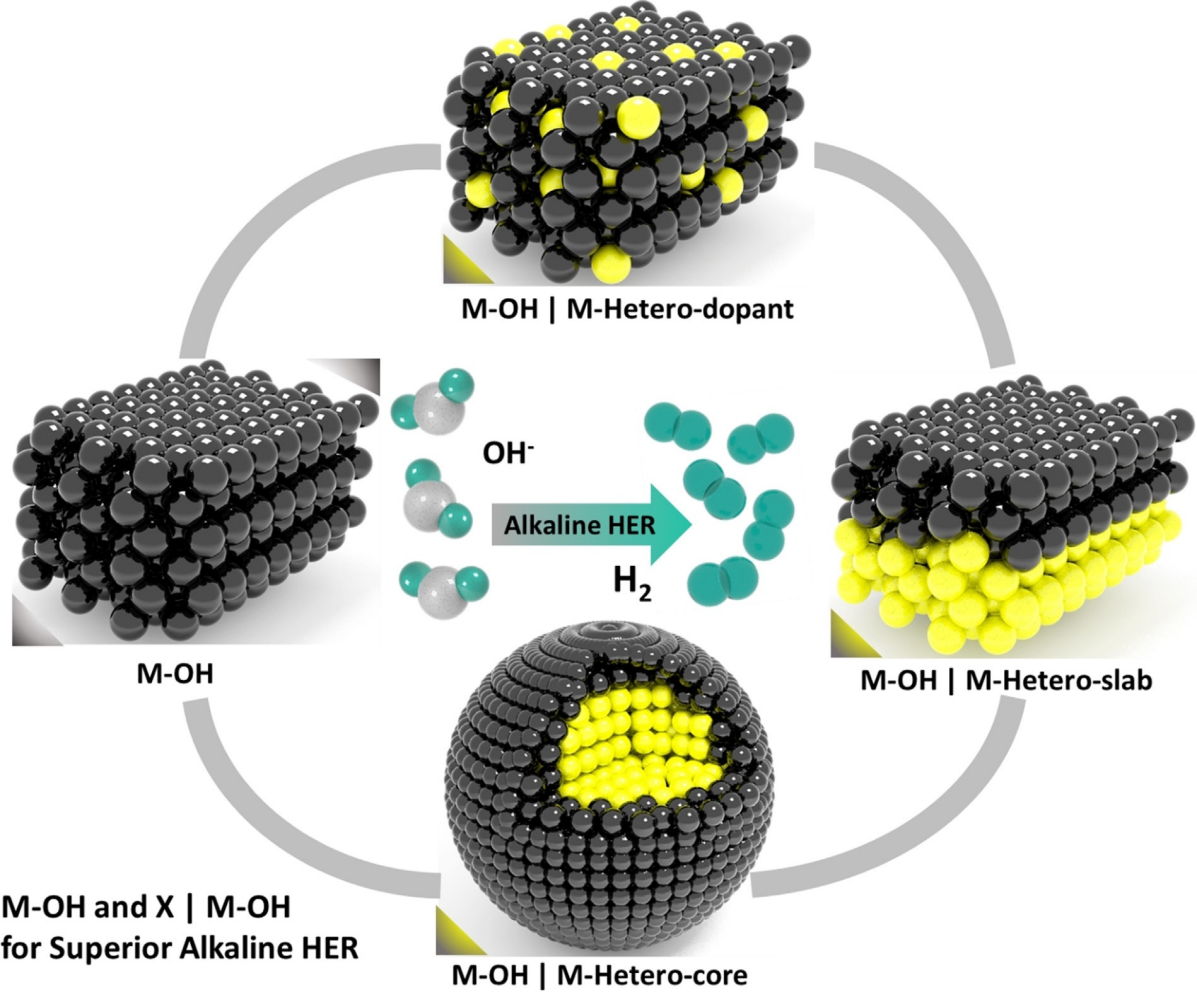
Graphical depiction of metal hydroxides and different types of heterostructured metal hydroxides used in alkaline HER.

Alkaline water electrolysis, in contrast, is blessed with a vast variety of non‐precious electrocatalysts for OER that include hydroxides/oxyhydroxides, oxides, chalcogenides, pnictides, carbides, borides and intermetallics.[^17^, ^28^, ^42^, ^43^, ^44^, ^45^] Among them, those non‐oxide/hydroxide catalysts are recently comprehended to act as precatalysts rather than actual OER catalysts because of the lability of non‐oxide/hydroxide anions that are easily displaced by hydroxide anion under highly alkaline solution.[^46^, ^47^, ^48^, ^49^] Such a displacement of non‐oxide/hydroxide anions occurs even under reductive potentials in alkaline solutions.[^50^, ^51^] Because of this phenomenon, it is now understood that most of the non‐oxide/hydroxide electrocatalysts that have so far been reported for alkaline HER must have catalysed it by forming a metal hydroxide phase on the surface that acted as the real‐time catalyst. Hence, it is explicit that for efficient alkaline HER, a metal hydroxide surface of an appropriate electronic configuration and moderate hydridic bond strength is inevitable.[^50^, ^52^, ^53^] The presence of lattice hydroxide anions is likely to enhance the water dissociation by facilitating the initial water adsorption with the partial negative charge that it possesses by hydrogen bonding.[^50^, ^52^, ^53^] Similarly, a relatively better HER performance witnessed with non‐oxide/hydroxide catalysts are likely due to the improved conductivity imparted by the conducting metallic and/or semiconducting phosphide/chalcogenide/carbide phases that also provide highly active defect sites that are formed by lattice mismatches at the junction of hydroxide phase and the heterophase which can be of several kinds in nature (Scheme 1).^[53]^ Perceiving the significance of metal hydroxides and their heterostructures (doped hydroxides, core–shell hydroxides, and hydroxides with slabs of hetero‐phases) catalysed alkaline HER in improving the energy and cost‐efficiency of alkaline water electrolysers, we believe that these catalysts are the missing pieces of the puzzle (i.e., energy‐efficient alkaline HER) that we have been dealing with. Further understanding and development in this area of electrocatalysis research, therefore, will ensure a comparable energy efficiency to that of PEM water electrolysers but fascinatingly with non‐precious catalysts which are and will be a great breakthrough in the field of water splitting electrocatalysis.

## 2 Alkaline HER: Why Is It So Hard?

Evolution of hydrogen gas from an aqueous electrolyte is largely pH dependent and is facilitated in solutions with a very high proton activity (i.e., acid solutions with pH 0). In such a highly acidic solution, regardless of the nature of the surface (metallic or semiconducting) that catalyse HER under applied potential, HER can proceed via two different paths namely Volmer–Heyrovský mechanism and Volmer–Tafel mechanism. In both paths, Volmer step is inevitable. Each step involved in acidic HER is given below [Eqs. 1, 2, 3].^[54]^

(1) $$\mathrm{Vomer}\;\mathrm{step}:\;\;H{}^{+}{}_{\left(\mathrm{aq}\right)}+e{}^{-}\to H{}_{\mathrm{ads}}$$

(2) $$\mathrm{Tafel}\;\mathrm{step}:\;\;H{}_{\mathrm{ads}}+H{}_{\mathrm{ads}}\to H{}_{2(g)}$$

(3) $$\mathrm{Heyrovsk}\overset{´}{y}\;\mathrm{step}:\;\;H{}_{\mathrm{ads}}+H{}^{+}{}_{\left(\mathrm{aq}\right)}+e{}^{-}\to H{}_{2(g)}$$

As can be witnessed, proton adsorption and discharge that forms adsorbed H atoms (H_ads_) would be facile in a solution of extremely low pH. The following step could either be Tafel or Volmer depending on the work function, density of states, and the local crystallographic geometry of the catalytic sites.[^55^, ^56^] In general, close‐packed systems with higher probabilities of forming more H_ads_ in closer proximities tend to evolve hydrogen following Tafel path and this is the most efficient path for HER that require minimum work (i.e., less applied potential).^[18]^ On the other hand, catalysts with widely dispersed catalytic sites in which the distance between two adjacent catalytic sites that could form H_ads_ is larger than the sum of Van der Waals radii of two S−H_ads_ bonds (S represents the catalyst site) tend to follow the Heyrovský step instead.^[8]^ This is an energy‐inefficient path when compared to the Volmer–Tafel mechanism. Hence, it is always desired to have HER catalysts that would follow Volmer‐Tafel mechanism. A simple steady state polarization curve analysis that helps us to calculate the Tafel slope will tell us the mechanism.^[57]^ Catalysts that have Tafel slopes closer to 30 mV dec^−1^ are said to follow Volmer–Tafel mechanisms and others with Tafel slopes higher than 45 mV dec^−1^ are said to preferably follow the Volmer‐Heyrovský mechanism. The selectivity between these two mechanisms shifts towards Volmer‐Heyrovský mechanism with the increasing Tafel slope until 120 mV dec^−1^. In acidic conditions, it is very rare to see a HER electrocatalyst that could have an abnormal Tafel slope (>120 mV dec^−1^) as the solution is rich in protons and the Volmer step is not hindered/dependent by any other reaction that must occur along. Nonetheless, it is also to be emphasized here that the Tafel analysis of HER in acidic conditions can be deceiving as proton discharge and the delivery of H_2_ molecule are very fast in proton‐rich solutions. Therefore, the measured Tafel slopes often reflect the mass transport limitations rather than reflecting the mechanism by which HER is being catalysed.

In alkaline conditions where the probability of finding a free proton in solution is nearly zero and the Volmer step (proton adsorption and discharge) has to rely always on a water dissociation reaction which is thought to be accompanied by the Volmer reaction and occurs separately prior to the Volmer reaction in the vicinity of electrode surface according to the beliefs of another part of researchers.[^50^, ^53^] Irrespective of when and how water dissociation occurs, the Volmer step of HER in alkaline solution is not identical to that of the one given in Equation (1) and so is the Heyrovský step as they both depend on water dissociation to form H_ads_/H_2(g)_. Here, the three steps of HER in alkali are listed below [Eqs. 4, 5, 6].

(4) $$\mathrm{Volmer}\;\;\mathrm{step}\;-\;H{}_{2}O\;\mathrm{Dissociation}:H{}_{2}O+e{}^{-}\to H{}_{\mathrm{ads}}+\mathrm{OH}{}^{-}{}_{\left(\mathrm{aq}\right)}$$

(5) $$\mathrm{Tafel}\;\mathrm{step}:\;H{}_{\mathrm{ads}}+H{}_{\mathrm{ads}}\to H{}_{2(g)}$$

(6) $$\mathrm{Heyrovsk}\overset{´}{y}\;\;\mathrm{step}\;-\;H{}_{2}O\;\mathrm{Dissociation}:H{}_{\mathrm{ads}}+H{}_{2}O+e{}^{-}\to H{}_{2(g)}+\mathrm{OH}{}^{-}{}_{\left(\mathrm{aq}\right)}$$

**Figure 1:**
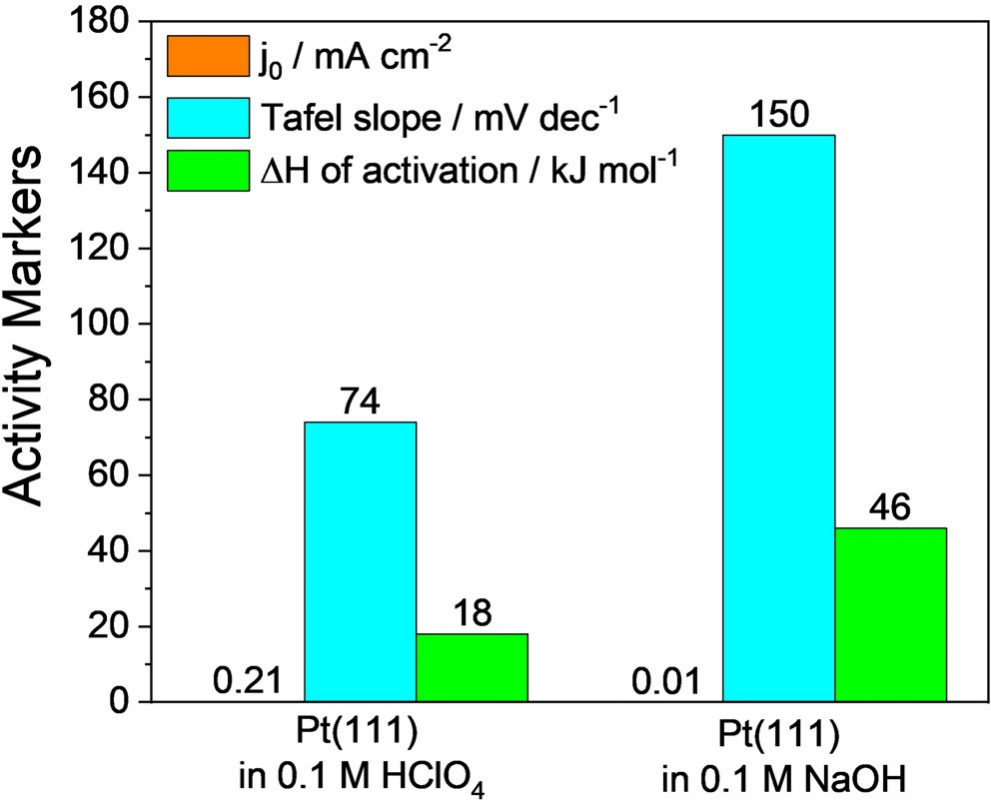
Exchange current densities (*j*_0_, in saffron), Tafel slopes (in cyan), and changes in enthalpy of activation (DH, in green) of Pt(111) surface in 0.1 M HClO_4_ and 0.1 M NaOH for HER. These values were taken from the studies of Markovic and co‐workers.[^59^, ^60^]

From the Volmer and Heyrovský steps of alkaline HER, it can be noted that the interface is given an extra burden to carry which is the water dissociation step providing the required free proton for the reaction to proceed. This implies that HER in alkali is indeed harder than acidic HER. In addition, it could also be noted that a hydroxide anion is produced locally at the surface of the electrode with the expense of a water molecule, which further makes things complicated as it will lower the concentration of water molecules and pushes the equilibrium of water dissociation back. These insights from the mechanism suggest that alkaline HER is an energy intensive process than acidic HER and even the state‐of‐the‐art Pt struggles to perform efficient alkaline HER.^[58]^ Figure 1, a bar diagram showing the values of exchange current density (*j*_0_) of HER/hydrogen oxidation reaction (HOR), Tafel slopes, and enthalpy changes (Δ*H*) of activation for a Pt(111) surface reported by Markovic and co‐workers.[^59^, ^60^]

As one can clearly see from Figure 1 that Pt(111) surface required higher enthalpy change for activation in alkali than in acid. Similarly, a massive increase in Tafel slope suggests that there was a change of mechanism of HER. A Tafel slope of 74 mV dec^−1^ implies that Pt(111) surface was following Volmer–Heyrovský mechanism in acid whereas an abnormal Tafel slope of 150 mV dec^−1^ imply that the Volmer step of HER is dependent on another reaction (water dissociation in this case) for it to proceed via proton adsorption and discharge. In alkaline conditions, it is very common to witness such high and abnormal Tafel slope values (>120 mV dec^−1^) that primarily indicate that the reaction is controlled by the water dissociation step and the rate determining step (RDS) is the first electron transfer reaction (i.e., the Volmer step). Hence, any HER electrocatalyst that could have a Tafel slope value lesser than 120 mV dec^−1^ is considered to be better in alkali and the catalysts that could follow the Volmer‐Tafel mechanism in alkali are rare which often contain Ru or Pt with metal hydroxide or heterostructured metal hydroxide phases.[^61^, ^62^, ^63^] Ru doped alkaline HER catalysts are thought to experience an enhancement in activity by the high affinity of Ru to dissociative complexation with water molecules that provides the required localized proton reservoir.^[63]^ On the other side, metal hydroxide phases with lattice hydroxide anion that are relatively deficient in electron population (as they are in the coordination sphere of metal ions) when compared to free hydroxide anion in the electrolyte are less likely to repel water molecules away and in fact, they are believed to attract water molecules closer to electrode surface via hydrogen bonding. Because of these facts, metal hydroxides, heterostructured metal hydroxides and metal hydroxides doped with Ru are performing better in alkali, particularly, the latter one outperformed Pt in alkaline HER.^[53]^ Hence, it is now explicit that for efficient alkaline HER, the metal hydroxide phase is inevitable.

## 3 Evaluation Perspectives: How Are HER Electrocatalysts Screened and Benchmarked?

**Figure 2:**
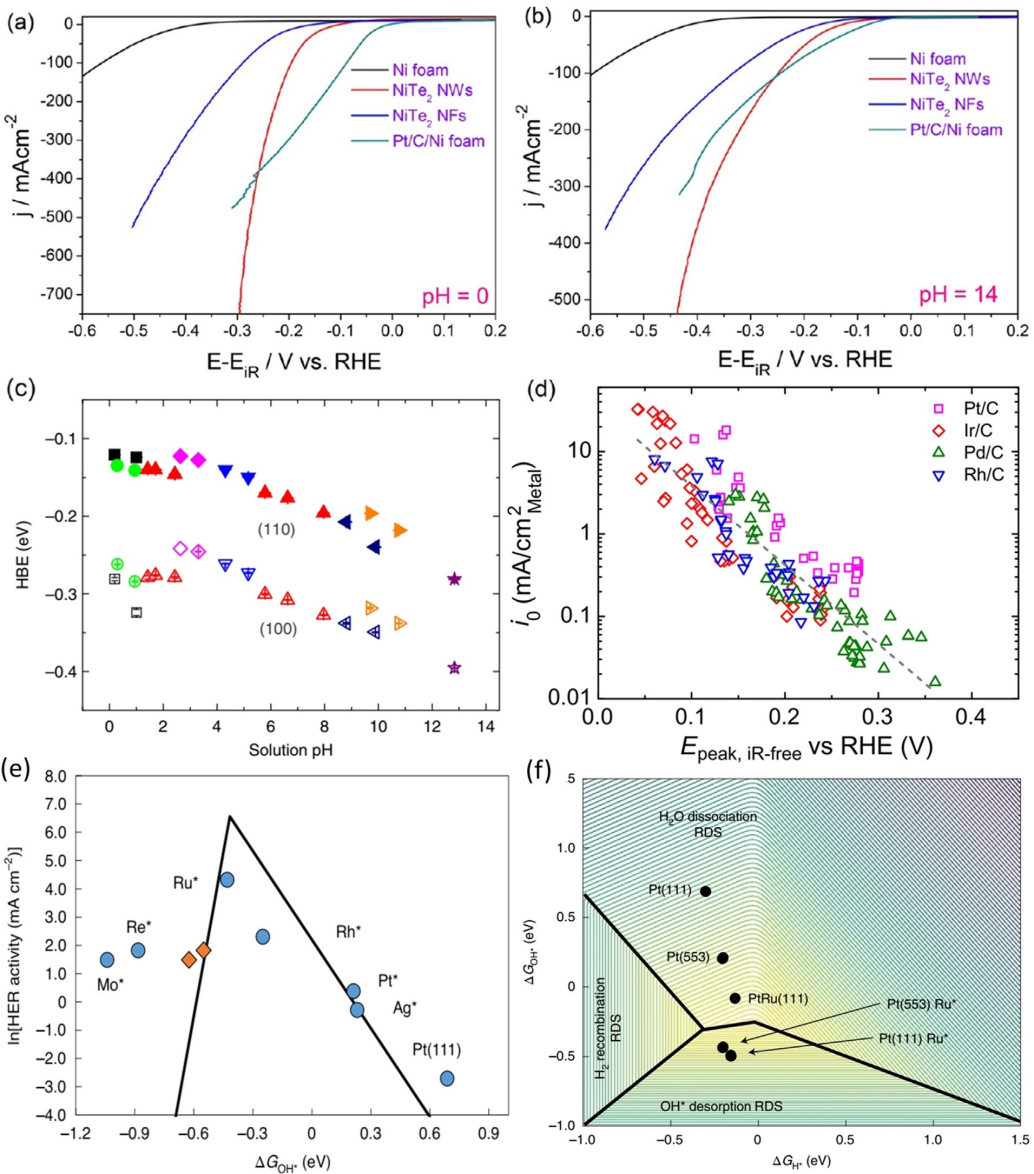
NiTe_2_ nanowires showing higher onset overpotentials but higher current densities at higher overpotentials when compared to Pt/C in acid (a) and alkali (b). Reproduced with permission from ref. [66] (Copyright 2018, ACS). (c) pH dependent hydrogen binding energy (HBE) on Pt(110) and Pt(100) surfaces.^[71]^ (d) A linear relationship arrived between the natural log of exchange current density and H_upd_ desorption peak for Pt group metals.^[72]^ (e) The plot of rate of HER of various metals decorated on Pt(553) single crystal electrode in alkali against the DFT‐calculated hydroxide binding strength at 0.0 V vs. RHE. (f) The 3D volcano relationship among the binding strengths of hydrogen and hydroxide ion with the energy of water dissociation for the single crystals Pt(111) and Pt(553), Ru decorated single‐crystal Pt(111) and Pt(553), and the alloy PtRu(111) showing that a weaker Δ*G*_H*_ and a stronger Δ*G*_OH*_ (at and around −0.3 eV) are essential for better water dissociation in alkaline HER. Both (e) and (f) are reproduced from ref. [74] (Copyright 2020, NPG).

As with all electrocatalysts, a HER electrocatalyst is also evaluated for high activity, selectivity, and stability which in combination with high abundance make it a perfect choice for energy and cost‐efficient hydrogen production.[^54^, ^64^] Activity is determined by both thermodynamics and kinetics of HER on a given electrocatalyst's surface. In general, an electrocatalyst is expected to catalyse the reaction of interest at the thermodynamically determined reversible/equilibrium potential (*E* ^0^) under ideal conditions. However, in real cases, there are other phenomena (mainly the kinetic complexities such as the water dissociation step in alkaline HER) that add further workload and makes the electrode to demand a certain quantity of additional energy (i.e. additional applied potential familiarly known as onset overpotential) to begin the reaction of interest. Beyond this onset overpotential, for every increase required in terms of current density will also increase the overpotential. Hence, it is desired to have a catalyst that could have lesser onset potential. An important characteristic of this onset overpotential is that it is intrinsic to the catalyst's surface properties and does not vary with the varying loading/specific surface area (SSA) of an electrocatalyst. This characteristic of onset overpotential places it among other important activity markers of a HER electrocatalyst. Nonetheless, there are several examples of electrocatalysts which were shown to have relatively higher onset overpotentials yet deliver better activity at higher overpotentials (current densities) due to various reasons among which increased SSA (thus electrochemical surface area (ECSA)) as a result of nanostructuring and increased loading are being the primary contributors to the overall enhancement witnessed.[^65^, ^66^, ^67^] An example is NiTe_2_ nanowires electrocatalysed HER in acid and alkali (Figure 2 a,b) which apparently required higher onset overpotential than Pt/C but outperformed the latter at higher overpotentials in current density.^[66]^ This implies that relying only on onset overpotential for determining the activity of an electrocatalyst could either overrate or underrate its actual performance at higher applied overpotentials, which is the case in real water electrolysers. Hence, researchers have adapted the practice of benchmarking these electrocatalysts at 10 mA cm^−2^ in analogy to the solar to fuel conversion (SFC) devices evaluation.[^64^, ^68^, ^69^, ^70^] For high‐performance catalysts, overpotentials at 100, and 500 mA cm^−2^ are also often reported along with onset overpotential and the overpotential at 10 mA cm^−2^. However, the recent comprehension is that any catalyst that is capable of delivering 1 mA cm^−2^ at lower overpotentials are considered better and they can be scaled up to improve their activity extrinsically depending on the requisites. Though onset overpotential and overpotential at any fixed current density (in cathodic direction) are primarily controlled and dictated by the hydrogen overpotential deposition (H_opd_), hydrogen underpotential deposition (H_upd_) was earlier thought to play crucial roles in determining the HER/HOR activity of electrocatalysts in water electrolysers and fuel cells. Hydrogen binding energy (HBE) of H_upd_ is highly pH dependent and it shifts toward more negative values (i.e., the corresponding potential will shift anodically) with increasing pH as reported by Yan and co‐workers for Pt surfaces (Figure 2 c).^[71]^ By this study, they revealed that such a change in HBE towards more negative values (i.e., the anodic shift of H_upd_ potential) is inversely related to the HER activity of Pt surfaces they studied. Laying the foundation on this finding, Zheng and co‐workers^[72]^ later came up with a more convincing way of determining HER activity of Pt group metals (Ir, Pd, Pt, and Rh) supported on conducting carbon by plotting the exchange current density (*j*_0_, the current density measured for HER at reversible overpotential) against H_upd_ desorption peak (Figure 2 d). From their study, it was found that catalysts with higher *j*_0_ and lower H_upd_ desorption potential (indicating weaker bonding of hydrogen on catalyst's surface) are highly active for HER. As indicated earlier, the hydrogen desorption/adsorption potential was found to shift anodically with increasing pH which justifies poor HER activity of electrocatalysts in highly alkaline solutions. Despite being an accurate activity marker, this relationship between *j*_0_ and H_upd_ desorption potential cannot be made for all electrocatalysts especially for semiconducting surfaces that hardly show such H_upd_ characteristics. This made this activity marker inapplicable for most of the recently evolved HER electrocatalysts. Besides, the HBE proposed by these studies is in fact inaccurate in alkaline solutions. This is because HER is pH dependent like H_upd_ but not Nernstian in nature like H_upd_. As a result, the guesses that could be made (for designing new catalysts) only from HBE may not be as impeccable as it was once expected to be in predicting the activity trends for alkaline HER. For example, the HBE was calculated to become highly negative for Pt with the increasing pH. However, the loss of HER activity by 2 to 3 folds in alkaline solutions was not justified.^[73]^ McCrum and Koper^[74]^ have very recently shown the importance of hydroxide binding strength (Δ*G*_OH*_) at the potential 0.0 V vs. RHE along with HBE for a catalyst to have a facile water dissociation step in alkaline HER. In this study, metals such as Mo, Re, Ru, Rh, and Ag were decorated on a Pt(553) single crystal electrode and their HER activity in alkali was screened. When the rate of HER was plotted against the Δ*G*_OH*_, it led to a classical Volcano relationship (Figure 2 e) in which Ru decorated Pt(553) occupied the atop position with the highest rate of HER and a stronger Δ*G*_OH*_. This study has proven that strength of both hydrogen binding and hydroxide binding are to be optimized for better water dissociation and an efficient HER activity in alkali. Specifically, a catalyst that binds hydrogen weakly and hydroxide strongly (at and around 0.3 eV) can act better in water dissociation step which is the bottleneck of efficient HER in alkali (Figure 2 f). Besides, overpotential at geometrical surface area normalized current densities, overpotentials at a defined mass normalized current density (mass activity), ECSA normalized current density (specific activity) are also used to precisely screen the catalyst under examination. A detailed discussion can be found in our earlier perspectives and reviews.[^8^, ^54^]

All those overpotentials mentioned above are thermodynamic parameters. On the other hand, activity is also controlled by the kinetic parameters. Two other main kinetic parameters that researchers use in HER electrocatalysis are Tafel slope and turnover frequency (TOF) in which the former one qualitatively suggests how faster the HER is occurring on the interface with certain values that are often closer to 30, 60, 90, and 120 mV dec^−1^.^[54]^ As far as the Tafel slope is concerned, the lower the value, the better the kinetics. Tafel slopes are inversely related to the charge transfer coefficient of the reaction which is a direct measure of how efficiently electrons are transferred across the interface. Besides, Tafel slopes also serve as a means to predict the mechanism that the interface follows. Any value closer to 30 mV dec^−1^ indicates that the interface catalyses HER by following Volmer‐Tafel mechanism whereas values higher than 60 mV dec^−1^ indicate that the interface is following the Volmer‐Heyrovský mechanism. The second kinetic activity marker, TOF is determined using the Equation 7 where *j* represents current density at a defined potential, *N*_A_ stands for Avogadro number, *n* indicates the number electrons transferred per molecule of hydrogen evolved (two in this case), *F* denotes the Faraday constant, and Γ indicates the number of active sites. Hence, the calculated TOF is expressed as the amount of hydrogen produced per second.

(7) $$\mathrm{TOF}=\left(j\times N{}_{A}\right)/\left(n\times F\times \Gamma \right)$$

Thus TOF simply tells us how quick an interface can be in catalysing HER under applied potential and serves as an important activity marker.

Selectivity and stability are two other important perspectives of HER electrocatalyst evaluation that determines the fate of the electrocatalyst under study in promoting it to large‐scale water electrolysers. Selectivity is defined as the percentage efficiency of an electrocatalyst in using the applied energy (potential/current) selectively for the desired reaction (HER in this case) which is commonly termed as Faradaic efficiency/coulombic efficiency.[^13^, ^14^] The most efficient way in determining coulombic efficiency of HER electrocatalyst is using gas chromatography (GC). Specifically, the amount of hydrogen produced under an applied potential is quantified using GC at regular intervals until five to six measurements are made. Then, these values are compared to the amount of hydrogen that is calculated theoretically using Faraday's law of electrolysis. Most of the reported HER electrocatalysts are shown to possess 100 % coulombic efficiency as there occur no competing reactions usually. Finally, the stability of a HER electrocatalyst is determined classically by two ways in which the first one is the static method. In this method, either a constant potential (chronoamperometry) or a constant current (chronopotentiometry) is applied and the change in current or potential is monitored with respect to time.^[70]^ This study is carried out at least for 12 h continuously and some do perform it for days. During the course of this study, degradation in activity is reported in percentage loss. The second method of stability evaluation is dynamic where a broad potential window covering various electrochemical features of the catalytic electrode including the HER region is chosen and a cyclic potential ramp is applied with a very high scan rate such as 100 or 200 mV s^−1^ and the number cycles may vary from a few hundred to thousands.[^14^, ^29^, ^54^, ^75^] Any change in onset overpotentials and in overpotentials at defined current densities is measured and reported. An ideal HER electrocatalyst is anticipated to show negligible changes upon such cycling. Therefore, a good HER electrocatalyst should have a lower onset overpotential, a lower overpotential at benchmarking current densities, a lower H_upd_ desorption potential (if the interface is a metal), and a lower Tafel slope. Similarly, exchange current density (*j*_0_), TOF, and coulombic efficiency should be higher. Meanwhile, the effects of substrate electrode's dimension and conductivity and the ways in which the catalyst is supported (i.e., drop‐casting, spin‐coating, sputtering, thermal evaporation, electrodeposition, hydro/solvothermal solution growth) should not be left aside. Depending on the conductivity and geometry of the substrate electrode, the observed activity of the same electrocatalyst can vary significantly. Such variations are highly pronounced particularly when the use of different substrate electrodes causes uncertainties in the catalyst's loading. Also, foam and fiber type 3D substrate electrodes often get access to the electrolyte beyond what is actually exposed via the capillary action. An elaborate discussion on the mesoscopic effects of substrate electrode geometry can be found in our earlier perspective,^[54]^ the review of Feng and co‐workers,^[76]^ and the viewpoint of Zheng and co‐workers.^[77]^

## 4 Alkaline HER and Metal Hydroxide Electrocatalysts

As stated earlier, metal hydroxides have recently been found to present at least on the surface of every non‐noble metals‐based HER electrocatalysts in alkali as a consequence of displacing action of highly electronegative, nucleophilic, and a relatively strong field ligand hydroxide.[^46^, ^47^, ^78^, ^79^] Thus it has now been doubted that each HER catalyst (except Pt, Au, and Hg) ever reported for an alkaline solution must have catalysed it by forming a secondary hydroxide phase at least on the surface. Perceiving this fact, we discuss here the critical advancements made in this field in arriving at such an understanding. Upcoming discussion will give a detailed account on the application of various metal hydroxides and metal hydroxide heterostructures for alkaline HER. Subsequently, their activities in terms of onset overpotential, exchange current density, Tafel slope, overpotential at 10 mA cm^−2^, and TOF are benchmarked while also indicating loading of catalysts which play crucial roles in altering overpotential at 10 mA cm^−2^ and TOF at the end.

## 5 Metal Hydroxides

**Figure 3:**
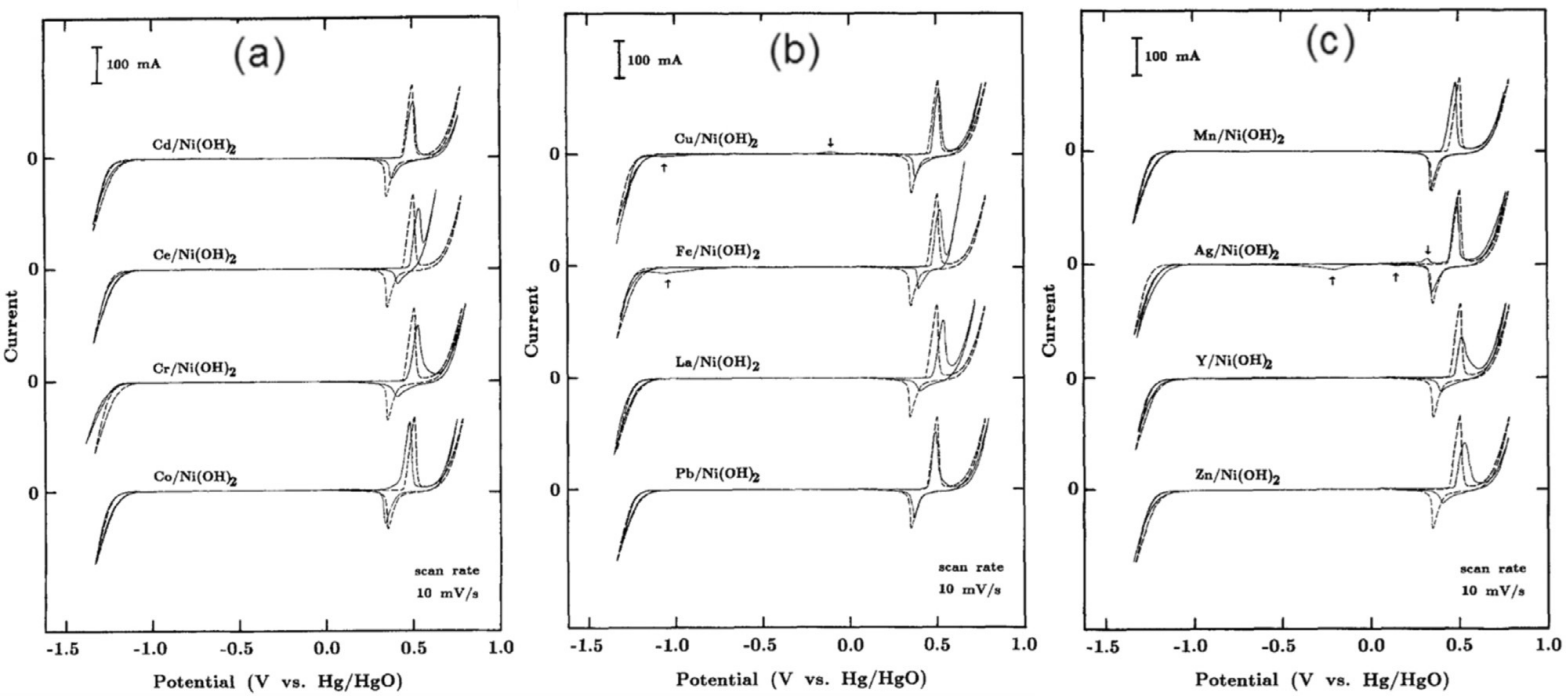
CV responses of Ni(OH)_2_ co‐precipitated with Cd, Ce, Cr, and Co (a), Cu, Fe, La, and Pb (b), and Mn, Ag, Y, and Zn (d) in 1 M KOH. Reproduced with permission from ref. [80] (Copyright 1989, The Electrochemical Society).

**Figure 4:**
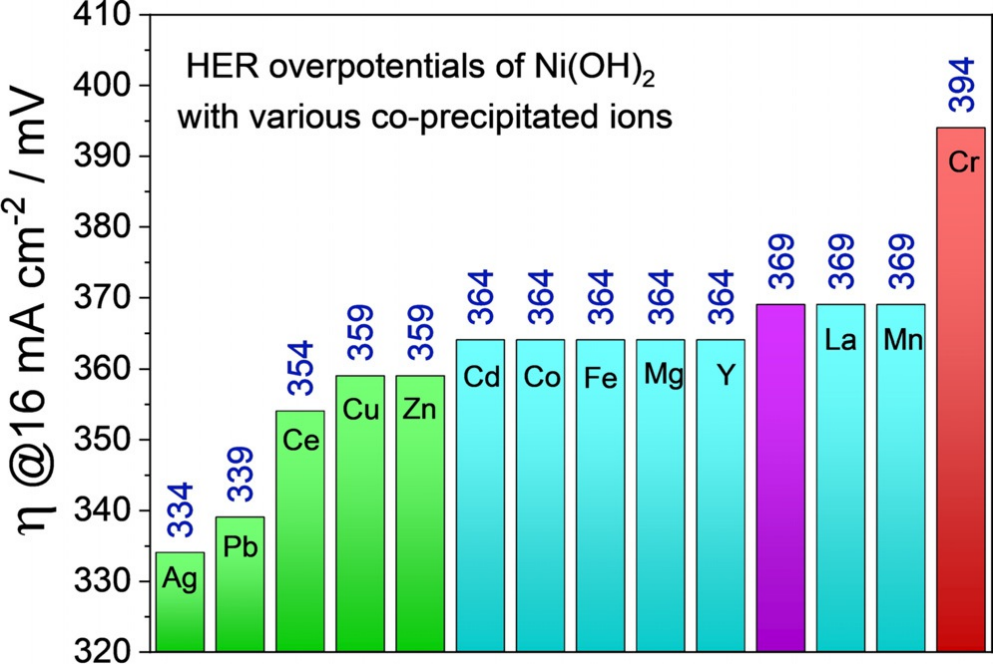
A bar diagram of HER overpotentials of Ni(OH)_2_ (purple) at 16 mA cm^−2^ in 1 M KOH with various catalytically enhancing (green) and poisoning (red) metal ions and also with metal ions (blue) that had played no significant role in changing its HER activity. All these values were taken from the study of Corrigan and Bendert.^[80]^

Alkaline HER electrocatalysts that contain mainly the hydroxide phase are frequently reported with commendable catalytic activity (see the benchmarking tables). These pristine metal hydroxides can have different crystallographic structures such as layered double hydroxides (LDHs), β‐hydroxides with a *hcp* arrangement, and multi‐metallic LDHs which is also called layered triple hydroxides (LTHs) when there are three different metals. However, LTHs vary in no crystallographic aspects when compared to LDHs. Nickel and cobalt hydroxides have also been shown to have appreciable HER activity in the alkali of which Ni(OH)_2_ always outperforms Co(OH)_2_ with a significantly higher margin in overpotentials. A very first and a detailed account on the HER (also on the OER activity) activity of Ni(OH)_2_ was reported by Corrigan and Bendert in the late 80s.^[80]^ This study revealed the effect of coprecipitating several rare earth, transition, and post‐transition metal ions (Cd, Ce, Co, Cu, Fe, La, Pb, Mg, Mn, Ag, Y, and Zn) with Ni(OH)_2_ on HER activity (Figure 3 a–c). Their key finding was that only Ag and Pb showed strong catalytic enhancement and Ce, Cu, and Zn showed a considerable catalytic enhancement in HER activity while the inclusion of other metal ions (Cd, Co, Fe, Y, La, and Mn) did not impart any notable changes in their HER overpotentials at 16 mA cm^−2^ (Figure 4).

Interestingly, coprecipitated Cr had poisoned the HER activity of Ni(OH)_2_ which is justified by its strong affinity towards forming the structurally rigid trivalent hydroxide. Chemistry of Pb and Ag are almost similar, they both have a similar density of states, both get precipitated with hydrogen sulphide, both form black oxides upon exposure to atmospheric oxygen in the presence of moisture. In contrast, none of the other metal ions studied along with these two have such similarities in their chemistry. Since then, advancements made with pristine hydroxide materials based HER electrocatalysts for alkaline electrolytes remained underdeveloped until the transfiguring nanostructuring of multimetallic hydroxide came into play. At the beginning of this decade, there was a renewed interest in re‐examining first‐row transition metal hydroxide for alkaline HER electrocatalysis. Hall and co‐workers^[81]^ in their study revealed that polished Ni electrodes kept at potentials corresponding to the formation of Ni(OH)_2_ for a longer time period gradually increased the HER activity which proved once again that a hydroxide phase of Ni is better active in alkaline HER than the fresh metallic surface of the same.

Later, hydroxides that had two or more metals were found to perform better than these monometallic hydroxides as they offer a variety of active sites with different but favourable hydroxide/water adsorption energies. This helped these catalysts to lower the threshold of Volmer step in alkaline HER.^[52]^ When binary metal hydroxides are considered, LDHs are the ones reported in the highest number for HER in alkali. So far, the reported combinations are Ni‐Co, Ni‐Fe, Co‐Fe, Ni‐Cr, and La‐Ni. In that series, Baranton and Coutanceu^[82]^ reported a very systematic study taking Ni and Co binary hydroxide system in which they varied the ratio of Ni and Co as 9:1, 7:3. 5:5, 3:7, and 1:9, respectively, and also compared the activity of only Ni and Co hydroxide. They made two significant observations viz., the Ni^2+^/Ni^3+^ redox couple was shifted cathodically with the increasing Co content and the same had also lowered the HER overpotential. Unexpectedly, only Co(OH)_2_ delivered better activity than both pure Ni(OH)_2_ and all other combinations of Ni and Co in the binary hydroxide they prepared. Despite the fact that a binary metal hydroxide with an optimal composition must show better HER performance than their monometallic counterparts, this observation stood out of the order. A careful observation on mass loading that they determined from TGA analysis showed clearly that Co was higher in loading (by weight percentage) than all other catalysts. This could be the reason for observing such a standout result. However, their study showed that Ni‐Co hydroxides of ratio 1:1 and 1:0.4 provided a better compromise between activity and stability. Following this study, Bai and co‐workers^[83]^ fabricated a Co‐Ni hydroxide composite on a 3D Ni foam that the benchmarking 10 mA cm^−2^ at just 77 mV from the reversible potential of hydrogen evolution. However, such an improved performance was obviously due to the 3D topology and huge mass loading of the catalyst which in turn was reflected by the huge double layer capacitance that they observed when compared to other catalytic interfaces taken along. A similar Ni‐Co LDH fabricated on Ni foam with a relatively lesser loading by Liu and co‐workers^[84]^ delivered poor activity by requiring 166 mV as overpotential for the same 10 mA cm^−2^. These two contradicting reports testify that besides intrinsic activity changes, mass loading, specific/ECSA, and mesoscopic effects play vital roles in determining the HER performances of these binary metal hydroxides. Zhao and co‐workers^[85]^ were the first ones to establish the mechanism by which Ni‐Co hydroxides perform HER in alkali. They termed it as the “pushing mechanism”. According to their study, the introduction of 10 % Co in Ni(OH)_2_ improves the HER performance by increasing the electron density at Ni centres via pushing the electrons that introduced Co had away from it when polarized cathodically. This pushing effect eased the Volmer step of HER and led to improved performance. On the other hand, when they added 10 % of Fe which longs for electron under applied potentials decreased the activity.

**Figure 5:**
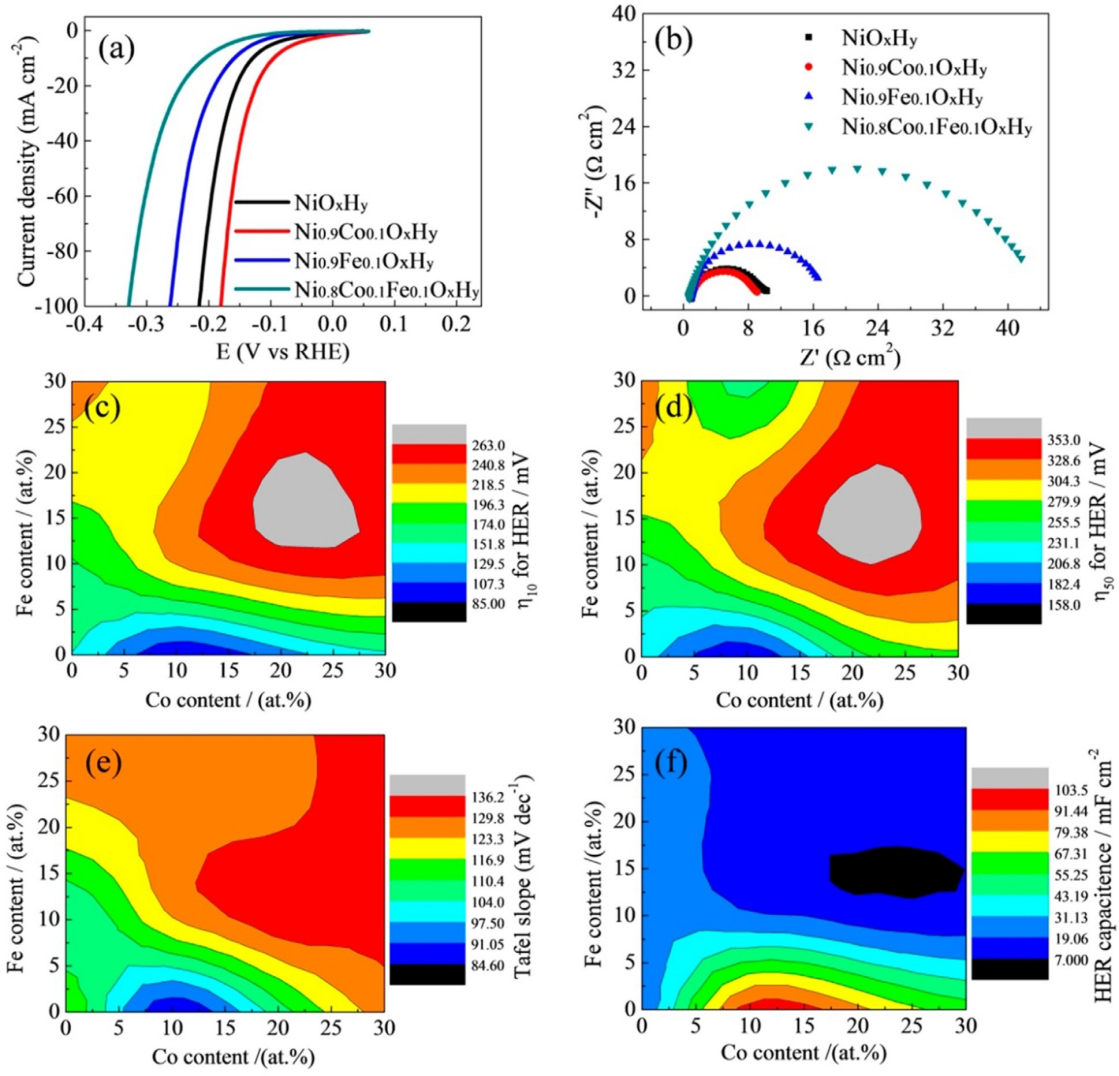
(a) HER LSVs acquire in 1.0 M KOH. Respective Nyquist plots (b) with contour maps of HER overpotentials at 10 (c) and 50 mA cm^−2^ (d), Tafel slope (e), and HER capacitance (f) of Ni(OH)_2_ catalyst with varying Co and Fe contents. Reproduced with permission from ref. [85] (Copyright 2018, ACS).

**Figure 6:**
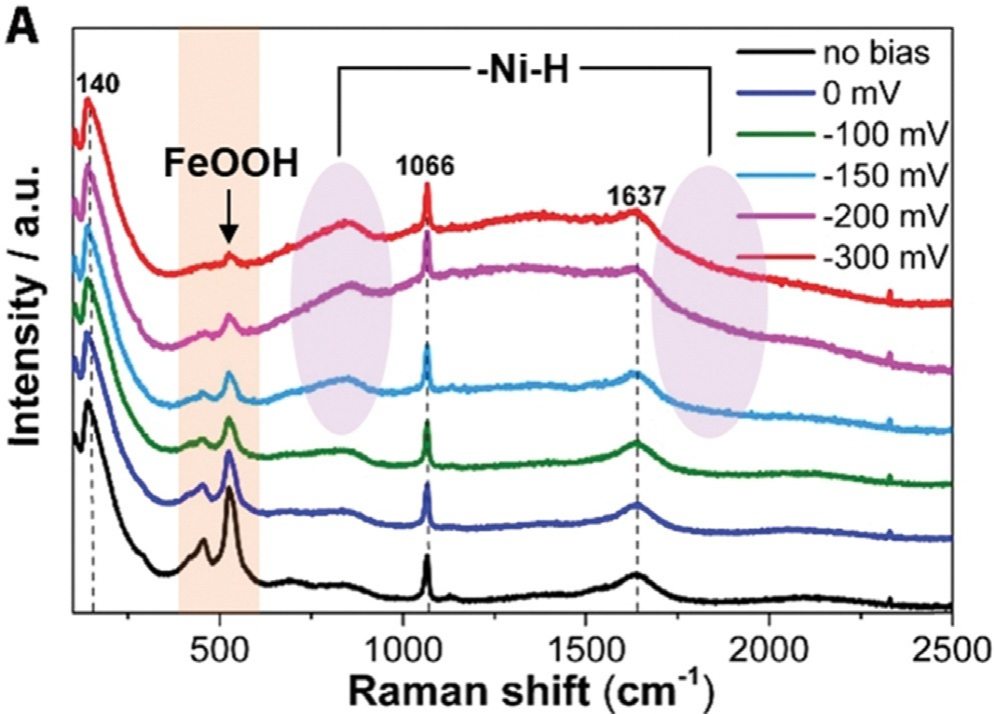
In situ Raman spectra acquired by increasing the cathodic overpotential with NiFe LDH HER catalyst showing the reduction of FeOOH peak and the emergence of Ni−H bonds. Reproduced with permission from ref. [90] (Copyright 2019, RSC Publishing).

Interestingly when both Co and Fe were added (20 % cumulatively) to Ni(OH)_2_, the HER performance was even worsened. These observations showed that a binary phase with a suitable metal with an appropriate electron donating ability is essential to observe a significant HER enhancement. Their observations on HER overpotential, Tafel slope, and double layer‐capacitance of Ni(OH)_2_ with different proportions of Co and Fe have indicated that Ni_0.9_Co_0.1_ hydroxide was the optimal system (Figure 5 a–f). Though the above‐discussed Ni‐Co binary hydroxide systems showed that the addition of Fe into Ni(OH)_2_ lattice would lower the HER performance, other researchers have shown contradicting results that are discussed below. One of such interesting results was shown by Rajeshkhanna and co‐workers^[86]^ who witnessed significant enhancement in the HER performance of NiFe LDH after performing chronopotentiometry (CP) stability test. However, in the same study, they concluded that before such CP testing, CoFe LDH was the better HER electrocatalyst which in turn was due to the ability of Co in donating electrons to water molecule with a lower applied cathodic potential. A similar CoFe LDH fabricated on 3D Ni foam by Babar and co‐workers^[87]^ did perform better in terms of overpotential (110 @ 10 mA cm^−2^) than the one reported by Rajeshkhanna and co‐workers^[86]^ which should have been the results of 3D configuration of the substrate electrode and high mass loading. Realizing the poor HER performance of NiFe LDH in alkali, Zeng and co‐workers^[88]^ in their similar work extended to perform total water splitting enriched the NiFe LDH phase with Ni(OH)_2_ and observed better activity. Nonetheless, the performance what they witnessed was still poorer than the ones attained with NiCo and CoFe LDH catalysts. Among the NiFe LDH based HER reports, Guo and co‐workers^[89]^ reported a systematic study by changing the molar ratio of Fe and Ni and the best activity was achieved with the catalyst which had 90 % Fe and 10 % Ni, which is very similar to the results that have been achieved with 90 % Ni and 10 % Co with NiCo LDH reported by Zhao and co‐workers.^[85]^ Here, the electron pushing element is Ni just like it was in the case of Co in Ni_0.9_Co_0.1_ LDH. However, given that Fe in 3+ state in NiFe LDH is more electronegative than that of Ni in 2+ state in NiCo LDH, the observed difference in HER performance between these two is justified. This fact was actually later supported by the in situ Raman study reported by Qiu and co‐workers.^[90]^ In this study, they found that with the increasing overpotential (towards the cathodic region), the Raman peak corresponding to FeOOH containing Fe^3+^ cation was diminished and led to gradually increasing HER performance as such reduction of Fe^3+^ progressed. They also revealed that alkaline HER with NiFe LDH begins with the adsorption of water at Fe^3+^ site as it is more electron deficient and proceeded further to form Ni‐H intermediate via a coupled proton‐electron transfer (CPET) mechanism. This Ni‐H intermediate then could be able to evolve hydrogen either via the Heyrovsky mechanism or Tafel mechanism (Figure 6). Other than Co and Fe, Cr and La have also been combined with Ni(OH)_2_ host to create an active HER electrocatalyst in an alkaline medium of which the NiCr LDH reported by Ye and co‐workers^[91]^ showed a substantially lower overpotential of 138 mV at a very high current density of 100 mA cm^−2^ when the ratio of Ni and Cr was 1:0.5. The observed abnormal HER performance was apparently high due to a huge mass loading (2 mg cm^−2^) rather than the intrinsic catalytic activity changes provided that Cr^3+^ does neither donate nor withdraw electrons easily as it is already having a half‐filled *t_2g_* level (in an octahedral field that exist in LDH phases) which is more stable than electron donating Co^2+^ and electron withdrawing Fe^3+^ in NiCo and NiFe LDHs, respectively. An unusual way of improving the HER activity of Ni(OH)_2_ was reported recently by Zhang and Hu^[92]^ which was the doping F that helped them to lower its HER overpotential by 91 mV at benchmarking current density (10 mA cm^−2^). However, the stability test performed for 12 h showed a significant increase in overpotential which is attributed to the leaching of F in alkaline medium just like any other halide ion does. Hence, this approach did not attract much attention lately to improve the HER activity of other similar HER electrocatalysts. Having a synergistic support with a HER electrocatalyst had always been shown to play benefitting roles.

Conductive and nanostructured carbons (graphene, graphene oxide, carbon nanotubes, etc.) with and without heteroatoms (N, O, F, S, P, B, and the combination thereof) are such proven synergistic supports in electrocatalysis research.^[93]^ Feng and co‐workers^[94]^ have shown for the first time that the HER performance of Co(OH)_2_ in alkali can be improved significantly by making a composite of Co(OH)_2_ electronically conducting polyaniline (PANI) fibres. This flexible electrode demanded less than 100 mV as overpotential to deliver 10 mA cm^−2^ for HER. Later, Jia and co‐workers^[95]^ achieved better HER performance with NiFe LDH in alkali by compositing it with defect rich graphene sheets. Interestingly, such a synergistic enhancement was also achieved with graphdyine with FeCo LDH by Hui and co‐workers^[96]^ which is the only HER electrocatalyst delivered current density as high as 500 mA cm^−2^ with lower overpotential. However, as there was no information on the loading of the catalyst, no justifiable comment or correlation can be made with the activity trend being discussed here. Similarly, Bhowmik and co‐workers^[97]^ reported HER activity enhancement in the case of CoFe LDH by compositing it with graphitic carbon nitride. These reports are among those examples for enhancing the HER performances of hydroxide electrocatalysts with carbon based synergistic supports. Besides compositing with a synergistically enhancing support material, enhancement in HER performances of these binary metal hydroxides was also achieved by defect engineering. Liu and co‐workers^[84]^ were the first ones to introduce this concept of defect engineering in HER electrocatalysis with CoFe LDH just by exfoliation in DMF‐ethanol mixture that helped them to realize improved activity. Very recently, Liu and co‐workers^[98]^ also showed that introducing O‐vacancy in CoFe LDH could improve its HER activity in alkali. These two studies are thus paving paths to engineer other metal hydroxides discussed above by introducing defects at metal centres or by making O‐vacancies which may improve their HER performances as these defects and O‐vacancies usually improve the electronic conductivity of these hydroxide materials because of the existence of unsaturated valances at metal and oxygen centres. From the above discussion, it can be concluded here that among all known monometallic hydroxides, Co(OH)_2_ is the best HER electrocatalyst and NiCo LDH takes that spot in the category of binary metal hydroxides‐based HER electrocatalysts because of the optimal electronic configuration of Co^2+^ ion that promotes the Volmer step.

**Figure 7:**
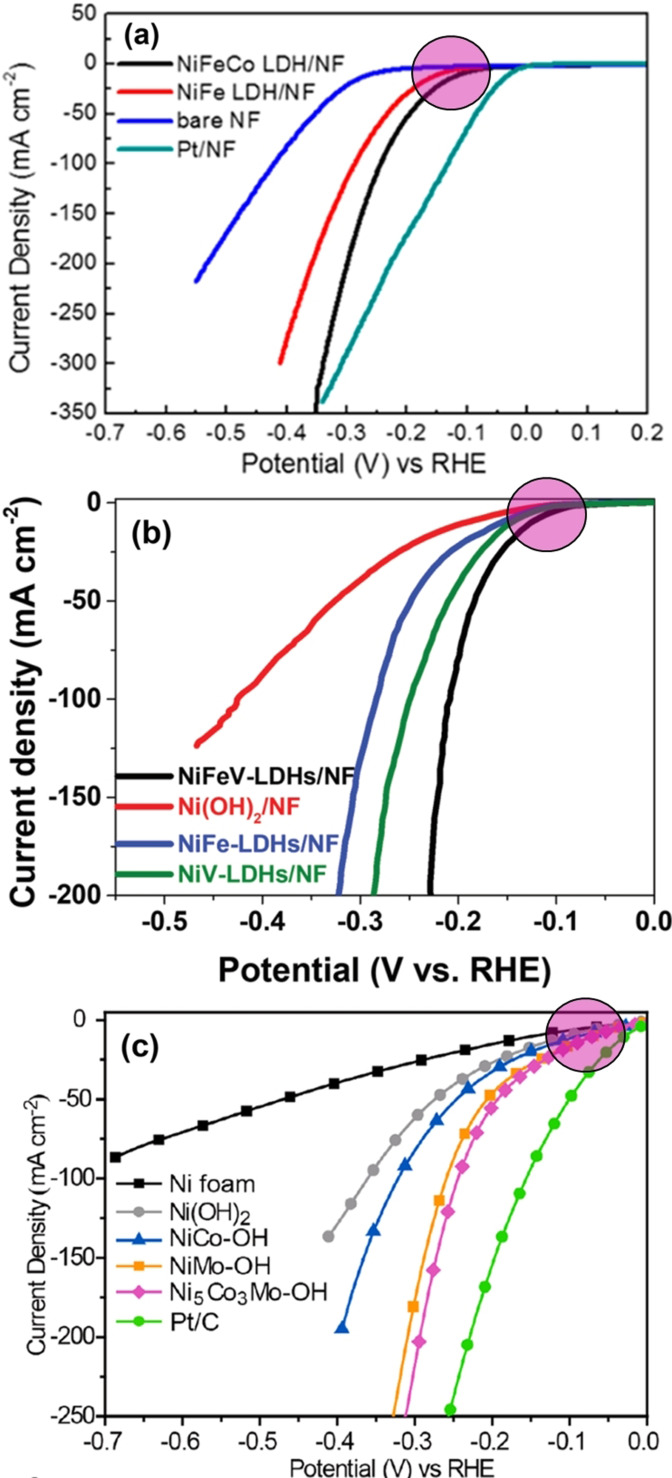
Trimetallic NiFe and NiCo LDH systems with a third metal (a) Co, (b) V, and (c) Mo showing no significant changes in the onset HER overpotential (indicated by pink circles). Reproduced with permission, respectively from ref. [101] (Copyright 2019, ACS), ref. [102] (Copyright 2018, Wiley), and ref. [103] (Copyright 2019, ACS).

Beyond just mono and binary metal hydroxides, researchers have introduced trimetallic hydroxides as HER electrocatalyst which are sometimes called a third metal doped LDH or directly as LTH. Such a concept was first introduced by Wang and co‐workers^[99]^ who made a NiCoFe LTH catalyst over carbon fibre cloth substrate that delivered better activity than all other control samples which included NiCo, NiFe, and CoFe LDHs too. Surprisingly, the activity shown for the best binary metal hydroxide (i.e., NiCo LDH) was very low in this report as we are not provided with the loading information here, no reasonable justification can be made. Zhu and co‐workers^[100]^ later revealed an optimal composition (Ni_2.5_Co_0.5_Fe) for the same system that performed relatively better albeit the differences were very small when compared with NiFe and Ni_2_CoFe systems. Later, Babar and co‐workers^[101]^ have reported the HER performance of NiCoFe hydroxide system in comparison with that of NiFe LDH (Figure 7 a). Though there was a significant improvement in overall HER activity, the retention of the same HER onset overpotential witnessed in this report implied that the observed enhancement was mainly due to the addition of more metal sites rather than intrinsic activity changes. In this series of trimetallic hydroxides, Dinh and co‐workers^[102]^ have introduced an untouched transition metal of the 3d series, the trivalent vanadium cation (V^3+^). This is yet another trivalent cation with a stable *t_2g_* level (empty) in an octahedral field of a LDH phase just like Cr^3+^ which also had a stable *t_2g_* level (half‐filled) in an octahedral field of a LDH phase. Hence, it did not impose any notable activity change when compared to the activity of NiFe LDH systems discussed above (Figure 7 b). This can be witnessed from the same HER onset overpotential that they reported for both NiFe and NiFeV LDH systems. Hence, the observed enhancement is mainly because of the added metal sites. The same trend and observation were made with the introduction of Mo into NiCo LDH systems too which was reported by Hao and co‐workers (Figure 7 c).^[103]^ All the above‐discussed metal hydroxide HER electrocatalysts are benchmarked based on their kinetic current density with respective mass loading wherever provided in Table 1.

**Table 1:** Benchmarking of metal hydroxide HER electrocatalysts based on the overpotential at 10 mA cm^−2^ in 1.0 M KOH.

Catalyst	Loading/ mg cm^−2^	Overpotential/ mV @ 10 mA cm^−2^	Tafel Slope/ mV dec^−1^	Reference
FeCo				
LDH@Graphdyine	N/A	50	98.9	Hui et al.^[96]^
NiCr LDH	2	52	61.5	Ye et al.^[91]^
Aged NiFe LDH	N/A	59	62	Qiu et al.^[90]^
Ni(OH)_2_/FeNi foam	N/A	70	160	Zeng et al.^[88]^
Co(OH)_2_@PANI	0.74	70	91.3	Feng et al.^[94]^
O‐Vacant NiFe LDH	N/A	73	69	Liu et al.^[98]^
Ni_5_Co_3_Mo‐OH	0.2	75	59	Hao et al.^[103]^
CoNi‐OH/Ni foam	N/A	77	94	Bai et al.^[83]^
Ni_0.9_Co_0.1_‐OH	N/A	77	90	Liu et al.^[104]^
LaNi LDH	N/A	80	60	Ensafi et al.^[105]^
F‐doped NiFe LDH	3.3	91	N/A	Zhang et al.^[92]^
NiFeCo LDH	N/A	108	73	Babar et al.^[101]^
CoFe‐OH/Ni foam	N/A	110	72	Babar et al.^[87]^
Ni_2.5_Co_0.5_Fe‐OH	0.25	110	93	Zhu et al.^[100]^
NiFeV LDH	1.42	125	62	Dinh et al.^[102]^
NiCo LDH	1	130	141	Liu et al.^[104]^
NiFe LDH with trace Fe	N/A	170	83	Rajeshkhanna et al.^[86]^
NiCoFe LTH	N/A	185	70	Dinh et al.^[102]^
CoFe LDH with trace Fe	N/A	205	93	Rajeshkhanna et al.^[86]^
NiFe LDH	N/A	210	78	Qiu et al.^[90]^
CoFe LDH @g‐C_3_N_4_	0.14	210	79	Bhowmik et al.^[97]^
NiFe LDH	N/A	220	74	Ye et al.^[91]^
NiFe LDH@DG	2	270	110	Jia et al.^[95]^
Defected CoFe LDH	0.2	300	95	Liu et al.^[84]^
NiCo‐OH	0.35	350	120	Baranton and Coutanceau^[82]^

Note: N/A stands for not available and implies that the corresponding data were not provided in the cited reports.

We urge the readers to be vigilant that this order should not be confused with the trend we discussed here as in some of the reports the loading information was not disclosed and the observed lower overpotential could be an effect a very high loading of the electrocatalyst.

## 6 Metal‐Metal Hydroxide Heterostructures

Metals have also been the first choice of electrocatalytic interfaces when it comes to HER in alkali as they were able to provide better electronic conductivity than oxides and hydroxides. Many early works have been concentrated mainly on Pt thin films, nanoparticles, and Pt/C composites.[^61^, ^62^] Other metals such as Ru, Ir, and Rh which showed a closer overpotentials for HER in alkali were also studied but not carried forward as one of the main objectives of switching water electrolysis from acid to alkali is to avoid these precious metals.

**Figure 8:**
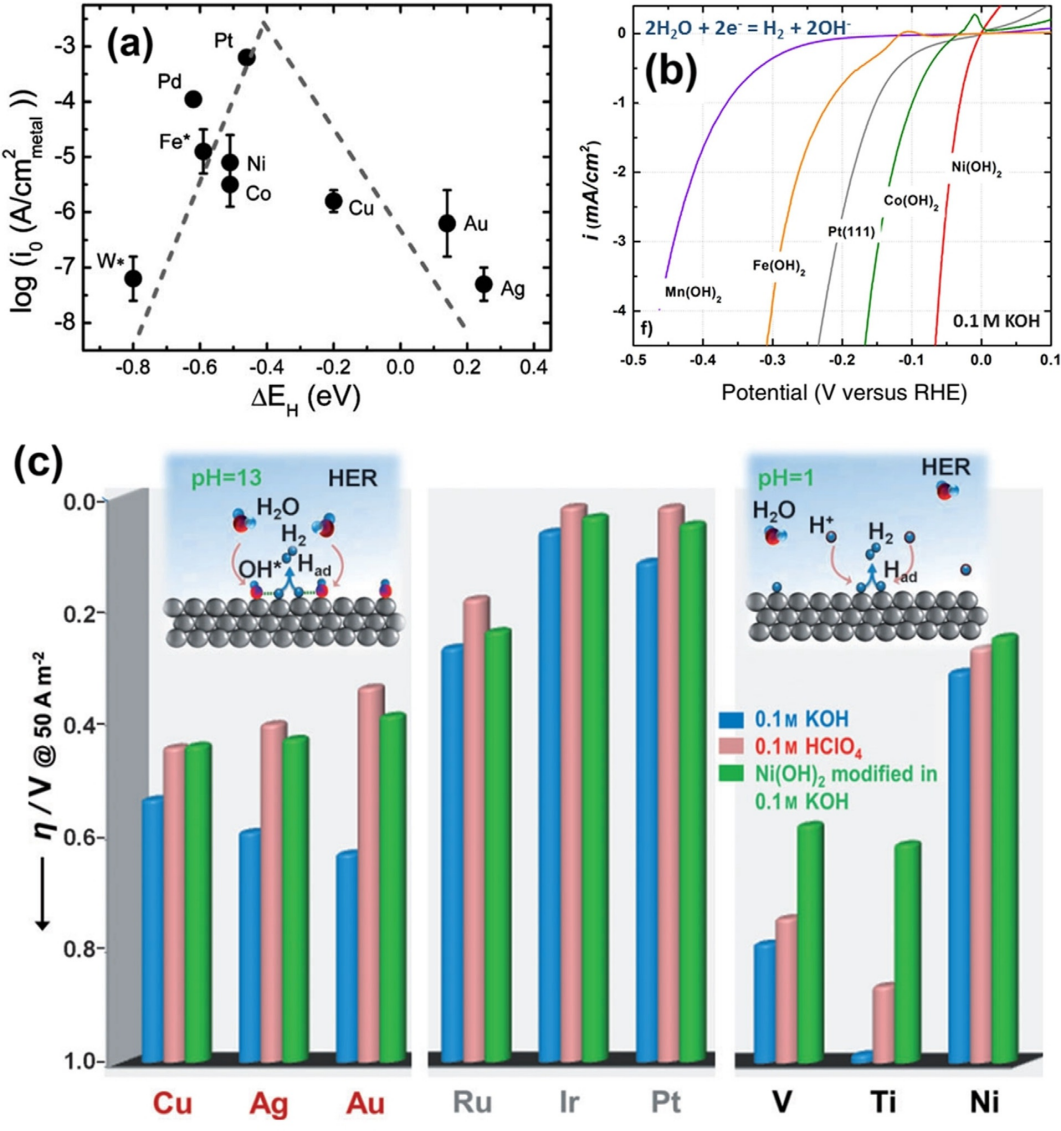
(a) Volcano plot of various HER catalysing metals. (b) HER LSVs of M(OH)_2_ (M=Mn, Fe, Co, and Ni) modified Pt(111) surface. (c) HER enhancing effect of Ni(OH)_2_ with Cu, Ag, Au, Ru, Ir, Pt, V, Ti, and Ni electrodes. All data were predicted and or acquired with 0.1 M KOH. Respectively reproduced with permission from ref. [106] (Copyright 2013, RSC Publishing), ref. [107] (Copyright 2016, Elsevier), and ref. [55] (Copyright 2012, Wiley).

**Figure 9:**
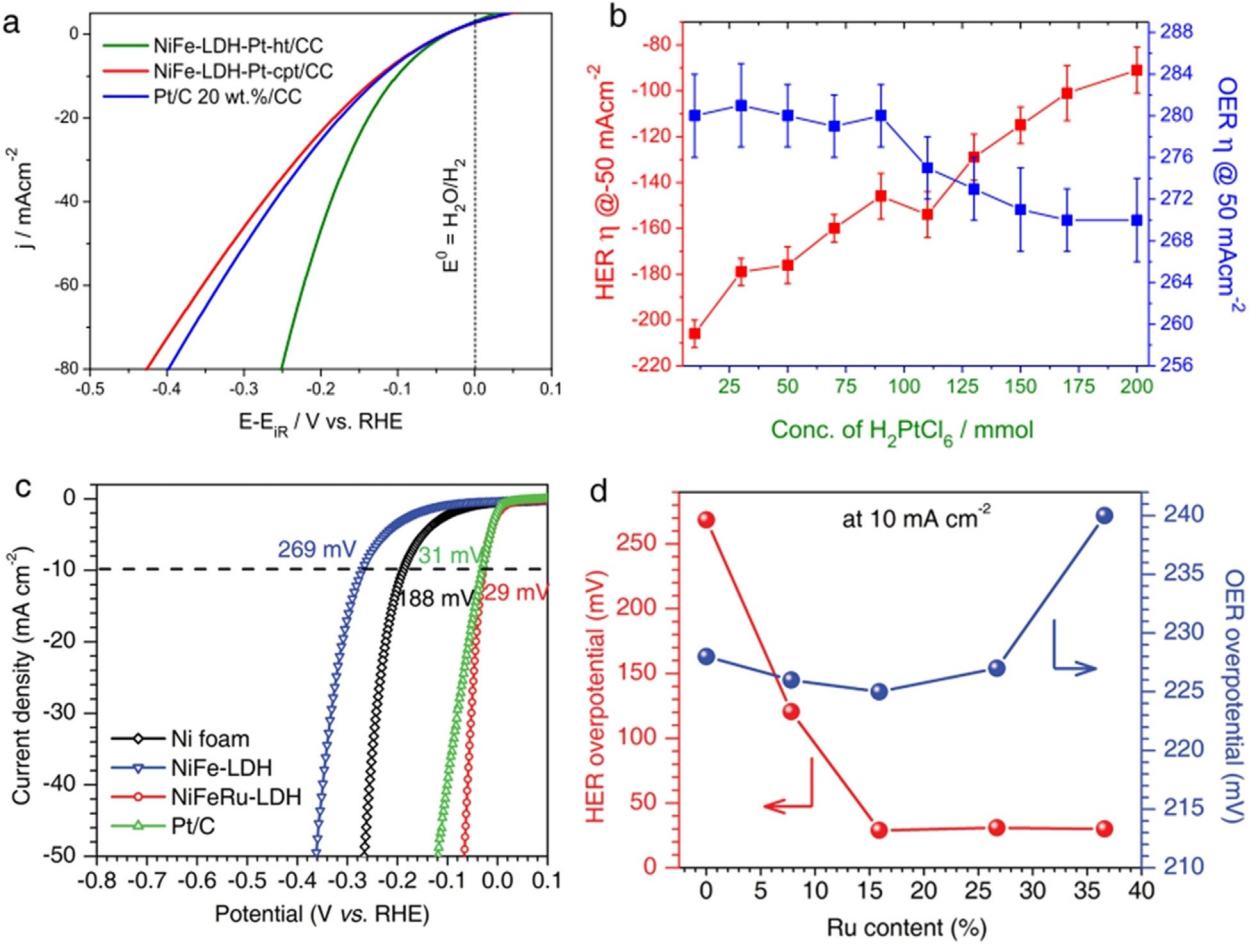
(a,b) HER LSVs of Pt‐decorated hydrothermally tailored NiFe LDH sheets and the effect of increasing Pt concentration. Reproduced from our earlier work.^[31]^ (c,d) A similar HER enhancing effect of Ru incorporation witnessed with NiFe LDH and the effect of increasing Ru concentration. The LSV of NiFeRu‐LDH shown in (c) is having 16 % of Ru. Reproduced with permission from ref. [116] (Copyright 2018, Wiley).

Sheng and co‐workers^[106]^ were the first ones to provide a generalized activity trend by plotting the exchange current density against the HBE which matched well with the experimental results of many metals (Figure 8 a). As anticipated, Pt stood at the apex of the volcano plot with an optimal exchange current density and HBE. Realizing that Pt(111) single crystal performs better than others, Strmcnik and co‐workers^[107]^ reported the HER activity trend of Pt(111)/M(OH)_2_ (M=Mn, Fe, Co, and Ni) heterostructures in 0.1 M KOH (Figure 8 b) which revealed that Co(OH)_2_ and Ni(OH)_2_ were actually enhancing the HER activity of Pt(111) surfaces whereas a significant loss of activity is witnessed with Mn(OH)_2_ and Fe(OH)_2_. This is justifiable given that Fe^2+^ in the low‐spin state has completely filled *t_2g_* level and Mn^2+^ high spin state has half‐filled *t_2g_* and *e_g_* levels in an octahedral field making them both stable. In other words, because of these stable electronic configurations, Mn^2+^ and Fe^2+^ ions tend not to donate or withdraw electrons which are necessary for drawing water molecules near hydrogen evolving metal sites. On the other hand, Ni^2+^ and Co^2+^ have unpaired electrons in their *e_g_* orbitals which are essential in deprotonating water molecules by donating them which in turn populate the nearby metal (Pt) site with necessary protons to evolve hydrogen efficiently. Between Ni^2+^ and Co^2+^, the former is better for enhancing HER as it does have two unpaired *e_g_* electrons. This also explains the exemplary HER activity of NiCo LDH materials among all other binary metal hydroxides. However, this does not mean that the electronic properties of the metal cations in the metal hydroxide phase alone contribute to alkaline HER enhancement. This was first shown by the work of Subbaraman and co‐workers.^[56]^ In this study, by incorporating dissolved Li^+^ cations into Ni(OH)_2_ layers that acted as a strong Lewis acid, they were able to achieve a further weakening of the H−OH bond in water. Such an additional H−OH bond weakening promoted the water dissociation step by Ni(OH)_2_ phase and subsequently enhanced its alkaline HER activity when interphased with Pt(111). Specifically, a 65 fold HER activity enhancement was witnessed with Pt(111)/Ni(OH)_2_ heterostructure when 0.001 M of Li^+^ cations were added to 0.1 M KOH.^[56]^ Hence, both interfacial and electronic effects are concluded to contribute together to enhance the water dissociation step for better alkaline HER. In contrast, the first ones to show such an HER enhancing effect of only Ni(OH)_2_ with various other metals such as Ru, Ir, Cu, Ag, Au, V, Ti, and Ni and including Pt earlier was Danilovic and co‐workers (Figure 8 c).^[55]^ In this study, they revealed that irrespective of the metal being studied, the heterostructuring of it with Ni(OH)_2_ had always resulted in an improved performance as it facilitates the dissociation of water which is the first and crucial step in alkaline HER. This was further confirmed by the work of Wang and co‐workers^[108]^ that showed the possibilities of optimizing the Volmer step by compositing Pt/C with single layer Ni(OH)_2_. Among all those metals, Pt performed better than others and showed parallel activity to that of Ru heterostructured hydroxides and hence, researchers were obviously attracted to study the Pt/Ni(OH)_2_ system more intensively than others by changing various structural properties of both Pt and Ni(OH)_2_ counterparts. Yu and co‐workers^[109]^ explored the difference in the HER enhancement brought out by the two most common phases of Ni(OH)_2_ that exist in alkali in the potential window of HER study. They found that the layered alpha phase was poorer than the rock‐salt structure having a beta phase as the latter one improves the kinetics of the Volmer step via a facile water dissociation mechanism. Since it is established that improved electrochemical access to a maximum of Ni(OH)_2_ in the Pt/Ni(OH)_2_ heterostructure is a key to improve the HER activity further, Yuan and co‐workers^[110]^ found that amorphous Ni(OH)_2_ decorated with Pt can deliver better HER activity as amorphous materials have always been benefitting water splitting electrocatalysis with improved ECSA. An interesting work of fabricating undercoordinated PtMn alloy and defect rich Ni(OH)_2_ heterostructure was made by Wang and co‐workers^[111]^ having defects and undercoordination helped them realize significant enhancement in activity. Particularly, the optimal heterostructure was found to have 0.7 wt. % of defect rich Ni(OH)_2_. Yu and co‐workers^[112]^ in a related work made a PtNi alloy that was heterostructured with Ni(OH)_2_ and when the PtNi:Ni(OH)_2_ ratio was 1:0.2, the best HER performance was achieved. Beyond just alloying, Pt was structured into different shapes at the nanoscale level as nanoparticles and nanowires and then heterostructured with Ni(OH)_2_ by Yin and co‐workers^[113]^ by doing which they witnessed better activity with Pt nanowires/Ni(OH)_2_ heterostructure. It was Ledezma‐Yanez and co‐workers^[114]^ who proposed the use of interfacial water reorganization as an appropriate descriptor for Pt/Ni(OH)_2_ catalysed HER in alkali using which Sarabia and co‐workers^[115]^ later showed the effect of interfacial water structure at Pt(111)/Ni(OH)_2_ heterostructure interface by varying the proportion of Ni(OH)_2_. They found that the increasing Ni(OH)_2_ proportion increased the HER activity here in this case too. Such enhancement was even highly pronounced when the flow‐cell was used which enabled relatively more access to interfacial water. However, McCrum and Koper^[74]^ recently showed (as discussed earlier) that more than interfacial water structure, the water dissociation step is crucial in determining alkaline HER activity. So far, the combination of Pt and Ni(OH)_2_ were only shown to be beneficial in alkaline HER, some of us recently discovered that a similar enhancement in HER can even be obtained with NiFe LDH by decorating Pt NPs. When the concentration of Pt precursor used to prepare Pt@NiFe LDH heterostructures was increased, the HER activity was also improved (Figure 9 a,b).^[31]^ Following this approach, Chen and co‐workers^[116]^ reported a similar HER enhancing with NiFe LDH by decorating Ru NPs instead of Pt. In their case, an increase in Ru content had led to further enhancement until the wt.% of Ru reached 16 % and no significant improvement was witnessed thereafter (Figure 9 c,d). In related studies, Li and co‐workers^[117]^ and Ma and co‐workers^[118]^ have recently shown that such HER enhancement can also be witnessed with NiCo LDH and cobalt carbonate hydroxide nanowires using Ru. Next to Pt and Ru, the most studied metal is Cu as a heterophase with metal hydroxide HER electrocatalysts in alkaline medium.

Yu and co‐workers^[119]^ showed that encapsulating Cu nanowires with NiFe LDH sheets could improve the HER performance of NiFe LDH significantly but the observed activity is nowhere closer to the ones observed with Pt or Ru interfaced heterostructures. The same group had also extended this strategy to CoFe LDH and observed a similar enhancement in the HER activity of CoFe LDH yet not comparable to those of Pt and Ru interfaced ones.^[120]^ Bai and co‐workers^[121]^ extended this strategy recently to Co(OH)_2_, Ni(OH)_2_, and as well to CoNi LDH and observed a similar HER enhancement. Other than Pt, Ru, and Cu, metals that were shown to bring out such HER enhancements were Mn with cobalt carbonate hydroxide, Ni with Ni(OH)_2_, Pd with NiCo LDH, Au with CoNi LDH, and W modulated Co(OH)_2_.[^122^, ^123^, ^124^, ^125^] Among them, W modulated Co(OH)_2_ was the only interface that delivered better HER activity than Pt/C.^[123]^ All these catalysts are benchmarked (in Table 2) by the overpotentials that they demanded at 10 mA cm^−2^ with different loadings and hence, we solicit the vigilance of the readers to not to take this order exactly as their actual HER activity trend.

**Table 2:** Benchmarking of metal‐metal hydroxide heterostructures based HER electrocatalysts as per the overpotentials they demanded for driving 10 mA cm^−2^ in 1.0 M KOH.

Catalyst	Loading/ mg cm^−2^	Overpotential/ mV @ 10 mA cm^−2^	Tafel Slope/ mV dec^−1^	Reference
Ru|NiCo LDH	0.7	28	42	Li et al.^[117]^
Pt|Co(OH)_2_	1	29	35.7	Zhou et al.^[124]^
Ru|NiFe LDH	N/A	29	31	Chen et al.^[116]^
Ru|Co‐(CO_3_)‐OH	N/A	66	65	Li et al.^[118]^
PtMn|Ni(OH)_2_	0.212	75	84.9	Wang et al.^[111]^
Pt|β‐Ni(OH)_2_	0.0013	92	42	Tang et al.^[126]^
Cu NWs|NiFe LDH	N/A	116	58.9	Yu et al.^[120]^
Cu|CoNi‐OH	N/A	150	48	Bai et al.^[121]^
Pt|Ni(OH)_2_	N/A	175	N/A	Wang et al.^[127]^
Cu|CoFe LDH	1.8	190	36.4	Yu et al.^[120]^
Mn|Co‐(CO)_3_‐OH	5.6	190	N/A	Tang et al.^[126]^
PtNi|Ni(OH)_2_	0.06	190	46.6	Yu et al.^[112]^
Au|CoNi LDH	N/A	210	92	Sultana et al.^[122]^

Note: N/A stands for not available and implies that the corresponding data were not provided in the cited reports.

## 7 Metal Oxide/Chalcogenide/Phosphide‐Metal Hydroxide Heterostructures

In this series of metal hydroxide heterostructures with metal oxides, mostly LDHs were studied with an oxide heterophase of the same metal ions or with different ones. The only study where a monometallic hydroxide/oxyhydroxide (Co(OH)_2_/CoOOH) was interfaced with an oxide phase of a dissimilar metal (PtO_2_ of Pt) was reported by Wang and co‐workers.^[128]^ In this study, it was shown that having Pt‐bonded O atom lowered the water dissociation free energy and improved HER performance. Besides, CoOOH in 3+ state here was actually playing the role of water abstraction site rather than regulating the Volmer step. Later, Wang and co‐workers^[129]^ showed that having a CeO_*x*_ heterophase with NiFe LDH could enhance its HER just by the way in which Co^2+^ acted by pushing the electron away from it to lower the energy of water dissociation as Ce in CeO_*x*_ existed in both 3+ and 4+ states. However, the magnitude of HER overpotential lowering is very low when compared to the overpotential lowering brought up by Co^2+^ in other systems. Another example of a metal oxide‐metal hydroxide heterostructure with partially different metal centres was reported by Wang and co‐workers^[130]^ who employed a series of hydrothermal and annealing methods to stack NiCo_2_O_4_ on Ni foam over which NiFe LDH was grown by using a hydrothermal reaction. The exact intension of this work is still elusive as neither NiFe LDH is a better HER electrocatalyst nor Co^3+^ ions in the spinel NiCo_2_O_4_ is capable of lowering water dissociation energy by donating electrons. However, the observed enhancement in HER activity was significantly higher and we believe that such enhancement could have possibly been brought by the in situ formation of Co^2+^ species under the applied reductive overpotentials. Nonetheless, no efforts were made in this work to find out any such in situ presence of HER enhancing Co^2+^ species.

**Figure 10:**
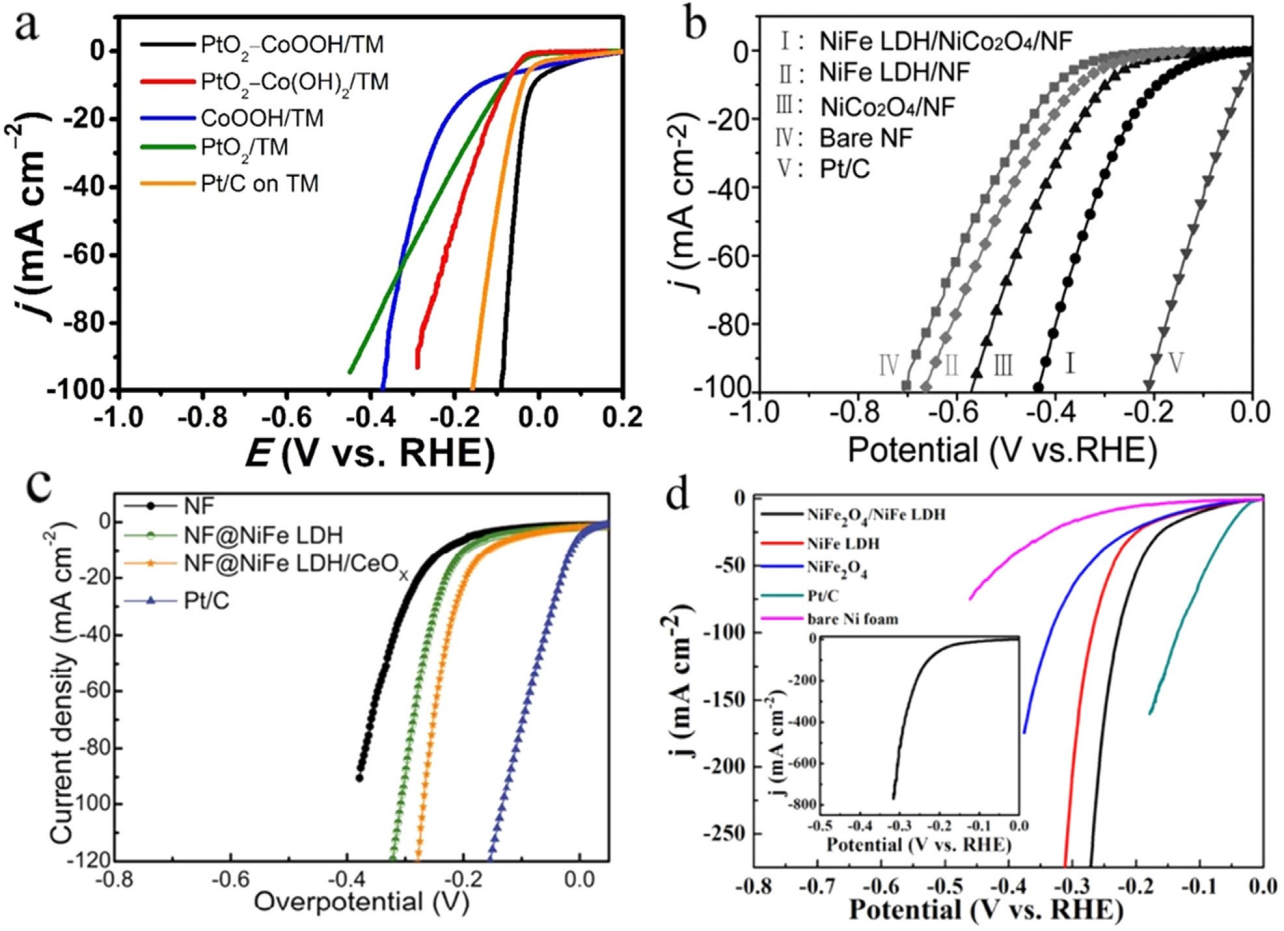
(a–d) Examples of HER enhancement witnessed by making metal oxide‐metal hydroxide heterostructures with similar and dissimilar metals. Respectively reproduced from ref. [128] (Copyright 2018, RSC Publishing), ref. [130] (Copyright 2017, ACS), ref. [129] (Copyright 2018, ACS), and ref. [131] (Copyright 2018, ACS).

However, Wu and co‐workers^[131]^ on the other hand, replaced this spinel NiCo_2_O_4_ with an inverse spinel NiFe_2_O_4_ and interfaced it with NiFe LDH which had electron donating Fe^2+^ ions in the tetrahedral field. As a result, a significant improvement in HER performance was witnessed. However, the lowering in overpotential was still higher with NiFe LDH‐NiCo_2_O_4_ catalyst reported by Wang and co‐workers.^[130]^ Around the same time, Li and co‐workers had shown a similar HER activity enhancement with NiCo_2_O_4_‐NiCo LDH. These studies have shown that not only a second metal of an appropriate electronic configuration or a metal heterophase but also an oxide heterophase of similar or dissimilar metals that could regulate the Volmer step can bring out notable enhancement in HER activities and imply the importance of having such heterostructures. Figure 10 a–d shows significant HER activity changes noted with such metal oxide‐metal hydroxide heterostructures.

**Figure 11:**
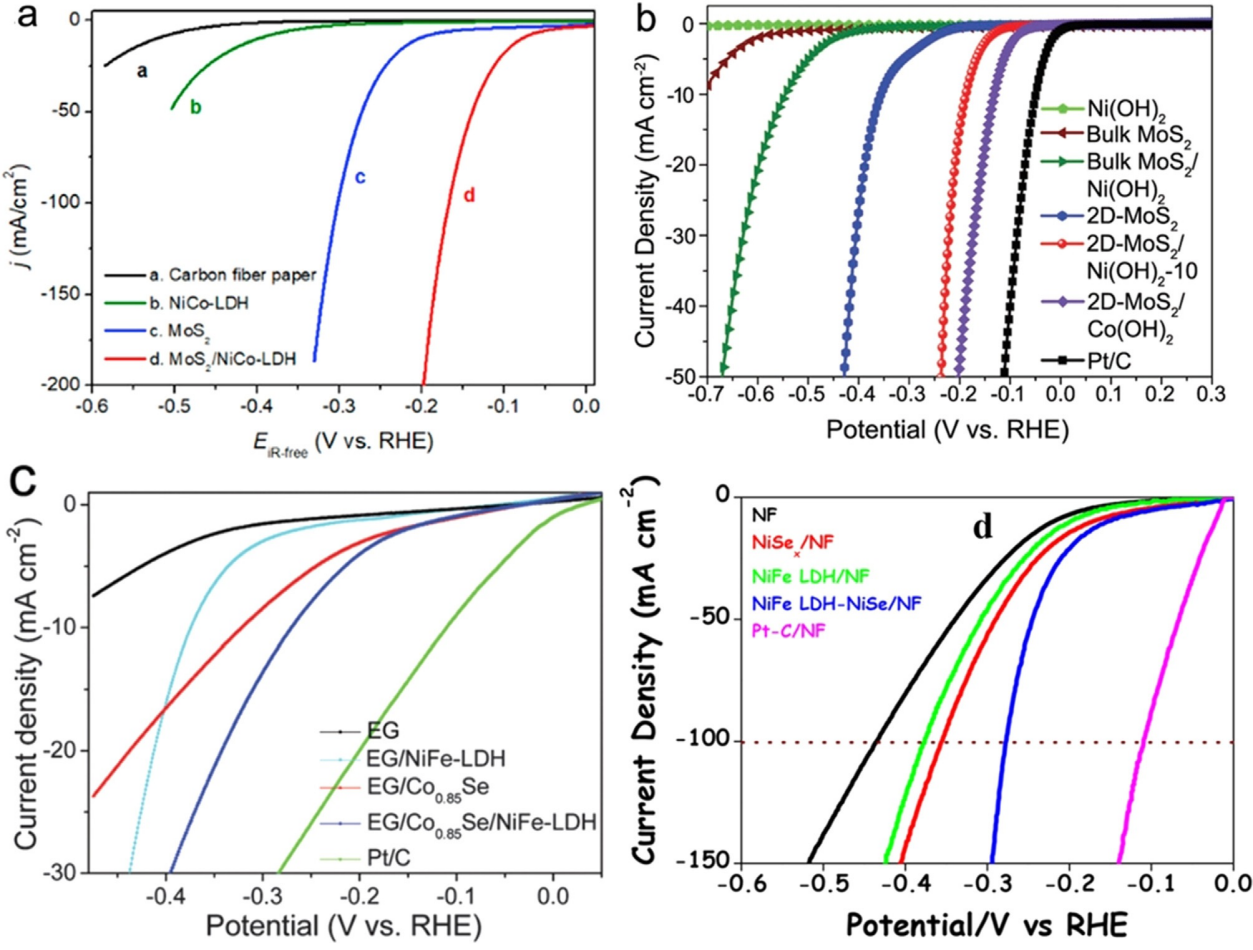
(a,b) Intrinsic HER activity enhancement realized by heterostructuring the best LDH (NiCo) and monometallic hydroxide (Co(OH)_2_) HER with metal chalcogenides reproduced, respectively from ref. [132] (Copyright 2017, Cell Press) and ref. [133] (Copyright 2018, Wiley). (c,d) HER enhancement witnessed because by heterostructuring CoSe and NiSe with NiFe LDH mainly by improved ECSA rather than intrinsic activity enhancement (indicated by the same HER onset overpotential and EG stands for exfoliated graphite) reproduced, respectively from ref. [135] (Copyright 2016, RSC Publishing) and ref. [136] (Copyright 2017, ACS).

Beyond metal oxides, chalcogenides of Co, Ni, Fe, Mo, and W were also interfaced with monometallic hydroxides and LDHs to realize efficient enhancement HER activity in alkali. Hu and co‐workers^[132]^ made first such efficient heterostructure by growing MoS_2_ on carbon fiber paper (CFP) followed by hydrothermally sandwiching the same with the best binary hydroxide (NiCo LDH) HER electrocatalysts. Activity enhancement brought up here were mainly due to intrinsic catalytic activity enhancement rather than a consequence of ECSA which was indicated by a large lowering HER onset overpotential and a marginal increase in double layer capacitance (Figure 11 a). Zhu and co‐workers^[133]^ in a related work modified the approach significantly. In this study, they exfoliated the established MoS_2_ and WS_2_ bulk materials and grew various monometallic hydroxides over exfoliated MoS_2_ and WS_2_ sheets and observed significant intrinsic activity enhancement with all of them among which MoS_2_ interfaced with Co(OH)_2_ (the best monometallic hydroxide HER electrocatalyst) delivered the best performance (Figure 11 b). Yang and co‐workers^[134]^ have later reported a similar intrinsic activity enhancement with CoNiSe_2_ interfaced with CoNi LDH (the best binary hydroxide HER electrocatalyst).

However, when either Co_0.85_Se^[135]^ or NiSe^[136]^ were interfaced with a relatively poor NiFe LDH, the activity enhancement was only marginal and was apparently due to the improved ECSA rather than intrinsic activity enhancement (Figure 11 c,d). In contrast, a contradictory observation with intrinsic activity enhancement with CoSe@NiFe LDH system recently reported by Sun and co‐workers^[137]^ stands out of the general trend that we have been showing through this review. In these cases of metal chalcogenide‐metal hydroxide heterostructures, metal hydroxide part performs the job of water dissociation (i.e., the Volmer step) and the metal chalcogenide surface catalyse the HER by forming hydridic intermediates. In this category of metal chalcogenide interfaced metal hydroxide, we have very recently reported an efficient way of making such heterostructures with an unconventional way.^[49]^ We subjected the NiS sheets grown hydrothermally over Ni foam substrate for anodic hydroxylation to form Ni(OH)_2_ heterophase rapidly. This method has additionally benefitted the HER activity improvement by increasing ECSA as a result of surface amorphization. As a result of collective benefit from both heterostructuring and amorphization, more than 100 mV of overpotential lowering was witnessed in alkaline HER at 100 mA cm^−2^. This method provides a facile path for heterostructuring other non‐oxide/hydroxide materials that can perform better in alkaline HER.

Metal phosphides interfaced metal hydroxide heterostructures are also reported to be a class of highly active HER electrocatalysts next to metal oxides and metal chalcogenides interfaced metal hydroxide heterostructures. Zhou and co‐workers^[138]^ had shown that making of CoNiP‐NiFe LDH could result in a substantial HER activity enhancement in alkali albeit the magnitude of enhancement was relatively low as expected because NiFe LDH is neither a good binary hydroxide HER electrocatalyst nor a cocatalyst that facilitate the Volmer step of the reaction. Xiong and co‐workers^[139]^ have purposefully formed a layer of Ni(OH)_2_ over Ni_2_P thereby forming a heterostructure witnessed a significant HER activity enhancement. They also revealed that the time of hydrothermal treatment and the associated thickness of Ni(OH)_2_ layer could influence the activity to a greater extent. Their results showed that a 10 h hydrothermal treatment was optimal in attaining the best HER enhancement out of this system. Zhang and co‐workers^[78]^ first showed that Co_2_P was prone to surface hydroxylation even under reductive potentials that degraded the activity. However, Su and co‐workers^[140]^ reported an important finding that anodizing CoP with 20 mA cm^−2^ could form undercoordinated Co(OH)_*x*_ species over CoP that helped them to realize a better HER activity. Like the metal chalcogenide interfaced metal hydroxide systems, metal hydroxides interfaced with metal phosphides (which are regarded as metallic in nature due to their high electronic conductivity) do also follow the same mechanism of HER where the hydroxide phase facilitates the water dissociation step and metallic metal phosphide perform HER by forming hydridic intermediates. All these catalysts are benchmarked taking the overpotentials that they demanded at 10 mA cm^−2^ in Table 3 and we also do remind here again that the readers should not adopt this order of benchmarking unless otherwise they are convinced with the loading of the catalyst material, ECSA, size, shape, and morphology.

**Table 3:** Benchmarking of metal oxide‐metal hydroxide, metal chalcogenide‐metal hydroxide, and metal phosphide‐metal hydroxide heterostructures based HER electrocatalysts as per the overpotentials they demanded for driving 10 mA cm^‐2^ in 1.0 M KOH.

Catalyst	Loading/ mg cm^−2^	Overpotential/ mV @ 10 mA cm^−2^	Tafel Slope/ mV dec^−1^	Reference
Metal oxide‐metal hydroxide heterostructures				
PtO_2_|CoOOH	0.056	14	39	Wang et al.^[128]^
NiFe_2_O_4_|NiFe LDH	2.8	101	67.1	Wu et al.^[131]^
NiCo_2_O_4_|NiCo LDH	N/A	115	56.4	Li et al.^[141]^
CeO_*x*_|NiFe LDH	N/A	154	101	Wang et al.^[129]^
NiCo_2_O_4_|NiFe LDH	4.9	190	59	Wang et al.^[130]^

Metal chalcogenide‐metal hydroxide heterostructures				
MoS_2_|NiCo LDH	N/A	78	76.6	Sun et al.^[137]^
CoSe|NiFe LDH	1.5	98	89	Sun et al.^[137]^
CoNiSe_2_|CoNi LDH	10	120	74	Yang et al.^[134]^
MoS_2_|Co(OH)_2_	N/A	150	76	Zhu et al.^[133]^
NiSe|NiFe LDH	2.01	170	70	Dutta et al.^[136]^
MoS_2_|Ni(OH)_2_	N/A	197	73	Zhu et al.^[133]^
Co_0.85Se_|NiFe LDH	N/A	260	160	Hou et al.^[135]^

Metal phosphide‐metal hydroxide heterostructures				
NiP|Ni(OH)_2_	1.26	43	58	Xiong et al.^[139]^
CoNiP|CoNi LDH	N/A	79	79	Zhou et al.^[138]^
CoP|Co(OH)_*x*_	N/A	100	76	Su et al.^[140]^

Note: N/A stands for not available and implies that the corresponding data were not provided in the cited reports.

All the above benchmarking tables (Table 1–3) display the catalysts in ascending order of the overpotentials that these catalysts demanded at 10 mA cm^−2^. This geometrical area normalized current density is highly dependent on the loaded mass of the catalyst.^[64]^ Loading is an important piece of information in all sorts of electrocatalytic studies including this HER which unfortunately is not disclosed in most of the reports discussed in this report.

**Figure 12:**
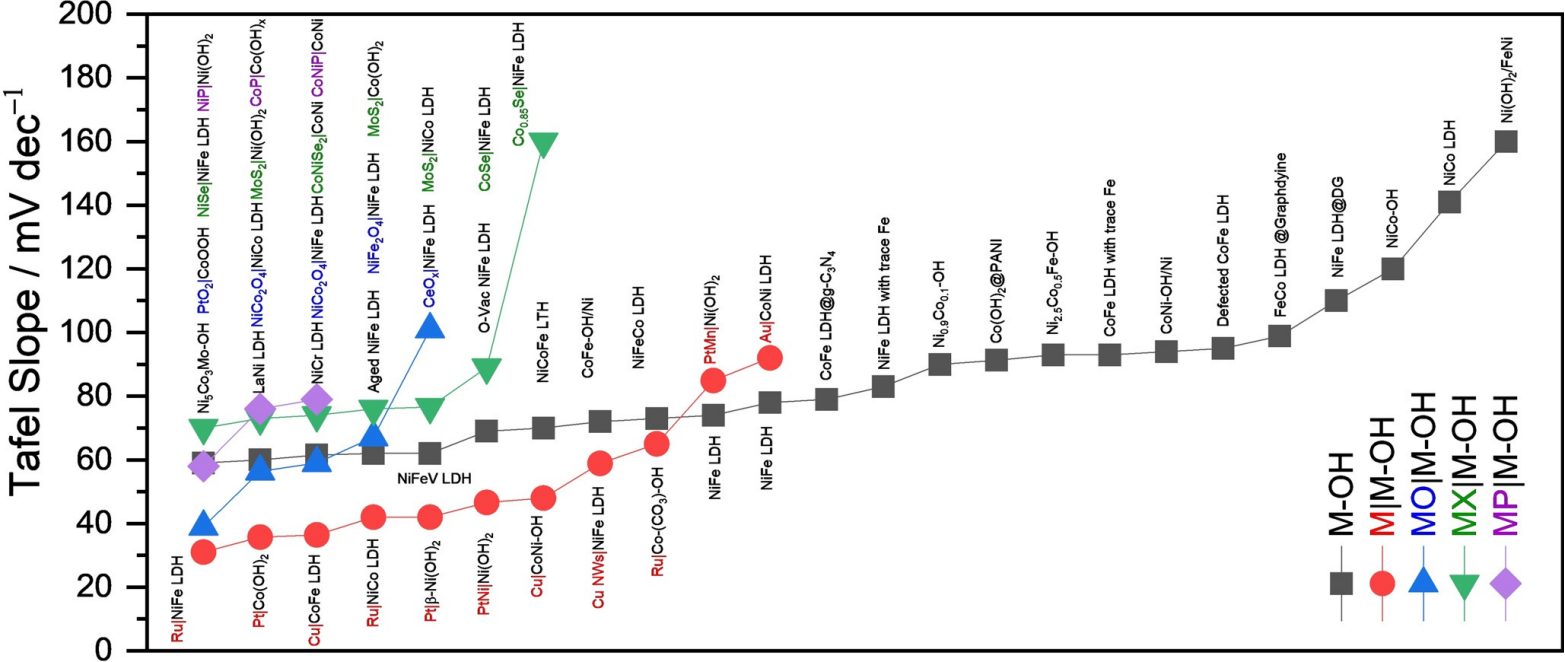
Benchmarking all the above‐discussed metal hydroxide and their heterostructures as HER electrocatalysts in an alkaline medium based on their Tafel slope values. (Note: For reference related to a particular catalyst labelled here please refer to their respective table and look for the same catalyst name).

This implies that the overpotential based benchmarking could be potentially wrong and misleading. Hence, to have an alternative view on the activity trends among all these catalysts, we portrayed all these catalysts discussed in each category according to their Tafel slopes (Figure 12) which primarily indicates the mechanism of HER and rate determining step (RDS). Since water dissociation coupled proton discharge is the RDS (i.e., Volmer step) of HER in alkaline medium and the Tafel slope of this step should in the range of 50–120 mV dec^−1^, any electrocatalyst that is having a Tafel slope ≤50 mV dec^−1^ can be considered a better active electrocatalytic interface. We have also shown in one of our recent studies that loading tends to affect Tafel slopes too but not as extensively as it does to the geometrical area normalized current densities and the corresponding overpotentials determined from it.^[64]^ From Figure 12, it is clearer that the Volmer step of alkaline HER is promoted highly with metal‐metal hydroxide heterostructures than other discussed ones and the pristine hydroxides. Specifically, the heterostructures of Pt, Ru, and Cu were shown to have the lowest possible Tafel slopes for HER in alkali. Therefore, we conclude here with the knowledge we acquired via an extensive literature survey that both metal NPs decorated metal hydroxide or metal hydroxide clusters deposited metal substrates can be efficient alkaline HER electrocatalysts. These are the ones that are so far shown to have demonstrated lower overpotentials and Tafel slopes in addition to better stability and selectivity.

## 8 Understanding Alkaline HER on Different Interfaces

**Scheme 2:**
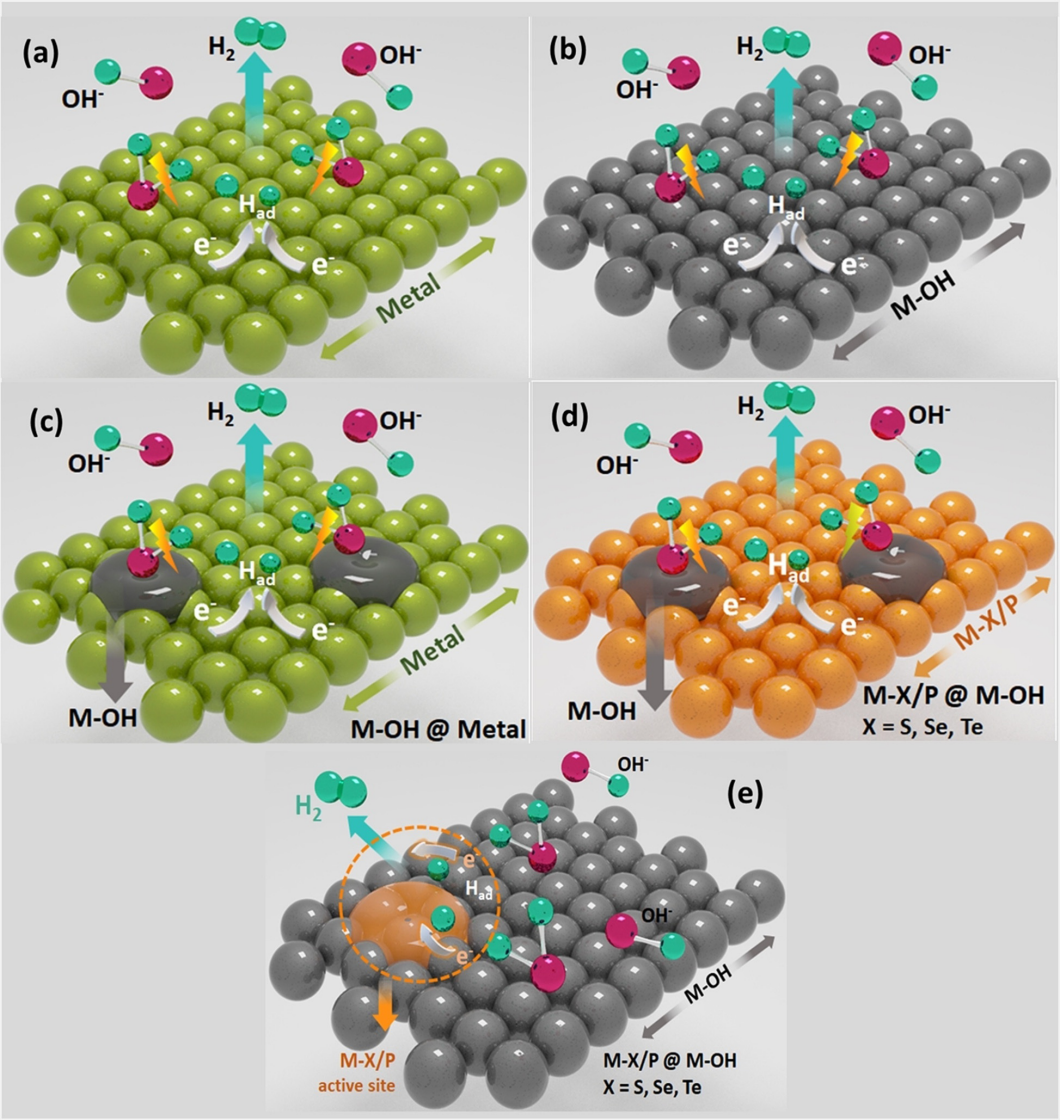
Illustration of water dissociation step's (Volmer step) site preferences on different interfaces in alkaline medium.

It is now certain that the efficiency of alkaline HER is greatly dependent on the efficiency of water dissociation into proton and hydroxide at the cathode which concurrently forms the hydridic intermediate with the catalytic site. This is the typical Volmer step of alkaline HER. This step of the reaction is so difficult as it produces more hydroxide at the interface which is already populated densely with it. Hence, the existence of a high concentration of hydroxide ions on the cathode surface pushes the water dissociation equilibrium backwards. From our ongoing discussions on the HER activity trends observed with metal hydroxides and their heterostructures, a graphical sketch (Scheme 2) is drawn illustrating which part of the catalyst tends to initiate water dissociation and which is efficient in making hydridic intermediate which then can evolve hydrogen molecule by following either Heyrovsky or Tafel mechanism.^[54]^ On the surfaces of metals that do not form any oxide or hydroxide under applied cathodic potential (e.g. Pt and Ru), water dissociation step is highly energy demanding and hence require high overpotentials to perform HER in order to achieve the desired current density. As a consequence of the fact that both water dissociation and hydridic intermediate formation ought to occur on the same surface metals usually perform poorly in alkaline medium. This is also the reason behind observing Tafel slope as large as 120 mV dec^−1^ and even higher. Metal hydroxide surfaces, on the other hand, are capable of facilitating the tedious water dissociation step because of their ability to coordinate reversibly with water and hydroxide ligands as both of them occupy closer places in the spectrochemical series. As a result, water molecules that coordinate with a metal cation site in a metal hydroxide surface can easily break the O−H bond of water when compared to the noble metal surfaces. This facileness of water dissociation with metal hydroxide surface enriches it with short living protons which can immediately undergo discharge to form the hydridic bond with a nearby catalytic site.

**Figure 13:**
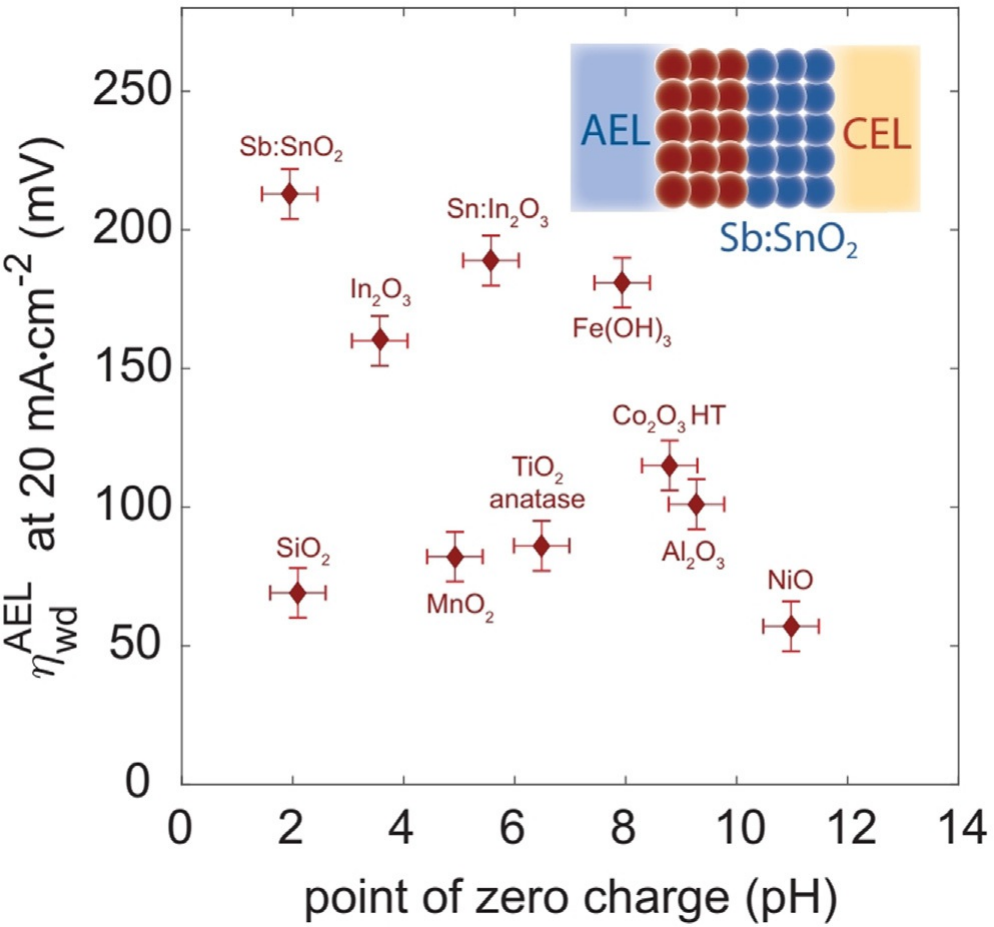
The relationship between water dissociation overpotentials at 20 mA cm^−2^ and point of zero charges (pzc) of various water dissociating catalysts placed on the alkaline side of a bipolar membrane showing a linear inverse relationship and signifying the importance of water dissociation step in efficient alkaline HER. Reproduced from ref. [143] (Copyright 2020, AAAS).

This catalytic site can be another metal cation in the same metal hydroxide phase or metals (Pt, Ru, Cu, Pd, W, Pd, etc.,), metal chalcogenides (of Ni, Co, Mo, W, etc.,), and metal phosphides (of Ni, Co, Mo, etc.,) that are interfaced with them. Among pristine metal hydroxides, Co(OH)_2_ is shown to be the better one as it dissociates water and concurrently forms hydridic bond by donating *e_g_* electron(s). In binary metal hydroxides, NiCo LDH is the best as they both have unpaired e_g_ electrons that facilitate both water dissociation and hydridic bond formation by “pushing” mechanism according to Zhao and co‐workers.^[85]^ When it comes to heterostructures of metal hydroxides, Ni(OH)_2_ heterostructured with Pt and Ru is the best as Ni(OH)_2_ splits O−H bond in water and the metallic counterpart (Pt or Ru) forms the hydridic intermediate concurrently which in turn facilitate HER. LDHs and other multi‐metallic hydroxides are also shown to exhibit similar activity trends when they are interfaced with Pt and Ru. However, Cu‐interfaced metal hydroxide and LDH heterostructures get benefitted from the better electronic conductivity of Cu. Hence, they impart better enhancement when they are sandwiched or wrapped around with a suitable metal hydroxide or LDH co‐catalyst. This is because metallic Cu tends to form Cu^+^ and Cu^2+^ species at potentials that are closer to HER and HOR when exposed directly to the electrolytes which cannot catalyse the Heyrovský and Tafel reactions efficiently. Besides, oxygen containing Cu^+^ and Cu^2+^ are well‐known for their poor HER activity which is one of several reasons why they are attractive for CO_2_ reduction.^[142]^ In a very recent systematic study by Boettcher and co‐workers,^[143]^ the predominance of water dissociation with NiO (which can form Ni(OH)_2_ in alkali) is ascertained once again to be controlling alkaline HER efficiency. This was revealed from the relationship between water dissociation overpotentials and the point of zero charges (pzc) of various water dissociating catalysts placed on the locally alkaline side of a bipolar membrane (BPM) (Figure 13). Catalysts with acidic and neutral pzc values did not show any rationalizable relationship with the water dissociation overpotentials. However, in alkaline conditions, NiO oxide with the highest pzc had the lowest water dissociation overpotential when compared to Fe(OH)_3_, Co_2_O_3_, and Al_2_O_3_. In the case of metal chalcogenides and phosphides interfaced metal hydroxide heterostructures, the hydroxide counterpart is predicted to perform the water dissociation step because of its ability to exchange water and hydroxide ligands easily.

It is recently comprehended that all non‐oxide/hydroxide HER electrocatalysts that were previously reported to be active in the alkaline media were actually forming a layer of hydroxide on their surface which in turn contributed significantly to the overall catalytic process.[^144^, ^145^, ^146^] Perceiving this, the research community has now started engineering the surfaces of such non‐oxide/hydroxide alkaline HER electrocatalysts purposefully to impart better activity. Examples are our recent work on NiS/Ni foam^[49]^ and the one by Su and co‐workers^[140]^ in which we employed anodic sweeping technique whereas they employed galvanostatic anodization that resulted in undercoordinated Co(OH)_*x*_ on CoP surfaces. Hence, it is concluded here that having a metal hydroxide heterophase with metal ions of appropriate electronic configurations (preferably with unpaired *e_g_* electrons) with known metal/metal chalcogenide/metal phosphide catalyst is the key to achieve better efficiency in alkaline HER.

## 9 Future Directions of Alkaline HER

**Figure 14:**
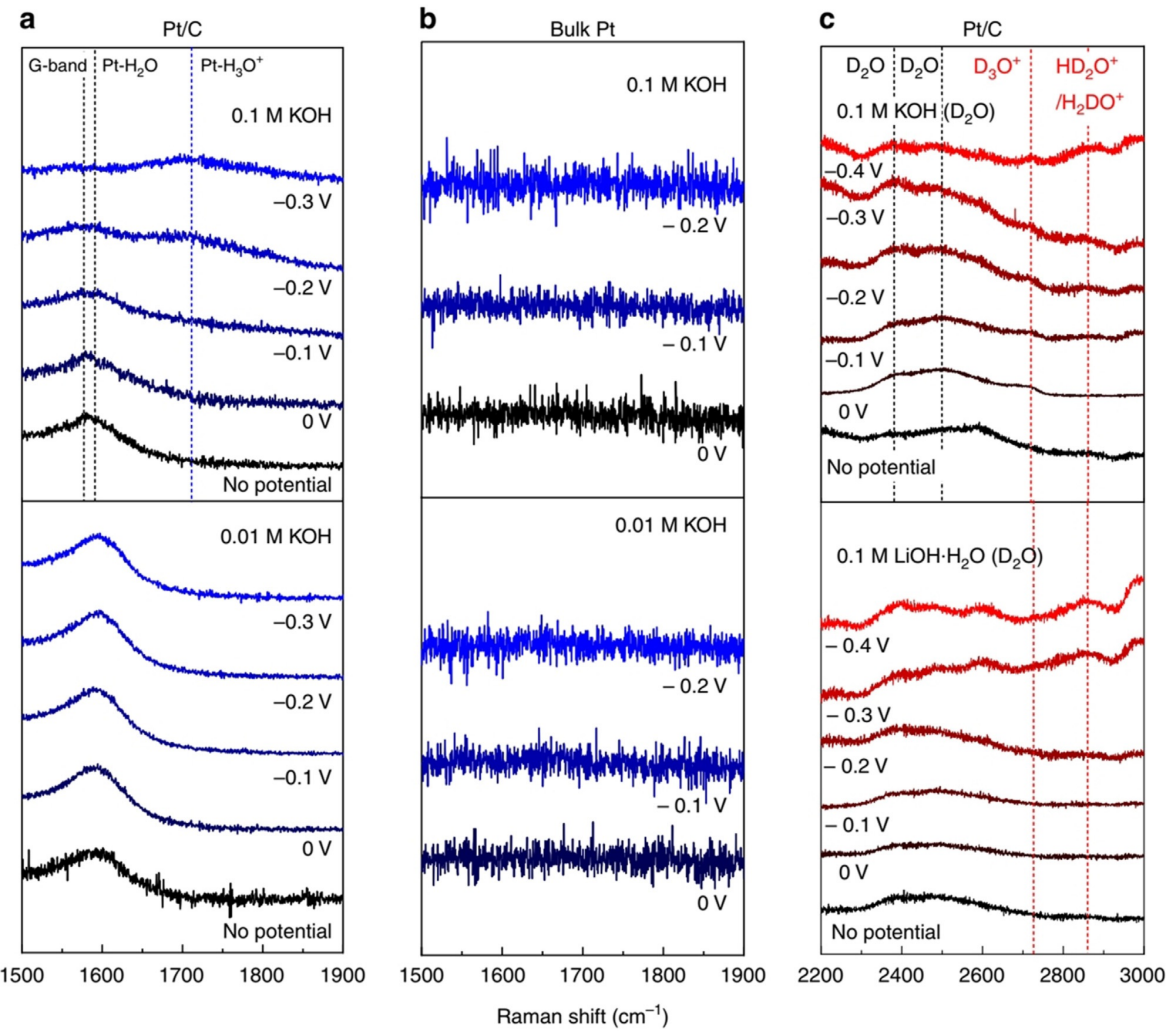
In situ Raman spectra acquired with Pt/C in water‐alkaline medium (a), with bulk Pt in water‐alkaline medium (b), and with Pt/C in the deuterated water‐alkaline medium. Existence of hydronium ion/protonated deuterium water or the combination thereof were witnessed only with Pt/C. reproduced with permission from ref. [150] (Copyright 2020, NPG).

As it is understood now that water dissociation plays a key role in determining the efficiency of an alkaline HER electrocatalyst, several works have now been focused on this particular part of the overall HER electrocatalysis. Meanwhile, it is also important to concentrate on the Heyrovsky and or Tafel steps. Although these two steps of HER primarily depend on the Volmer step, the bond strength of hydridic intermediate formed with the catalytic site also matters significantly. In our earlier review,^[8]^ we have provided an elaborated overview on the bond strengths of H to various elements that compose most of the known HER electrocatalysts.^[8]^ For a facile Heyrovsky and or Tafel step, it is essential to have a moderate S−H bond strength. In this point of view, Pt and Ru from the metals and P and Se from non‐metallic counterparts of the metal chalcogenide and phosphide catalysts have moderate strength. Hence, we expect that future research will be concentrated on these elements for quite a long time until a further breakthrough occurs. A list S−H bond strengths can be found elsewhere for the appropriate design of HER electrocatalysts.[^147^, ^148^, ^149^] In situ and operando spectroelectrochemical characterizations have been of tremendous use recently in understanding the structure of catalysts under operating conditions at various applied potentials and also in tracking the structure of electrical double layer and the species that exist during electrocatalysis.^[44]^ Since alkaline HER electrocatalysis is widely accepted to begin with the water dissociation step that provides the necessitated protons at the cathode surface, it is also essential to study the interface and electrical double layer simultaneously to know more about this widely‐accepted mechanism. An interesting and insightful study was reported recently by Qiao and co‐workers^[150]^ on the HER electrocatalysis of Pt in alkali using in situ Raman spectroscopy. They found that when Pt/C was subjected to catalyse HER at high overpotentials, due to a vigorous water dissociation, a highly acidic localized environment in the vicinity of the electrode was generated which successively increased HER activity. The same was confirmed using deuterated water and alkaline medium too (Figure 14 a–c). This effect was observed only with the nanostructured Pt/C but not with the bulk Pt indicating that the size and shape of electrocatalysts can have huge impacts on the way in which HER is performed. This study also implies that such in situ or *operando* spectroelectrochemical studies of alkaline HER with all the above‐discussed metal hydroxide catalysts and their heterostructures may even lead us to achieve fascinating results and could improve our understanding. Meanwhile, they could also prove our current understanding of alkaline HER be incorrect and provide other exciting insights. Hence, we sincerely hope that these analytical tools will play critical roles in the advancement of alkaline HER electrocatalysis. Other than this, the performance of every catalyst under a large A/V condition (large electrode surface area (A) with smaller electrolyte volume (V)) at elevated temperature which is the typical industrial condition may vary significantly.^[151]^ Hence, it will be inevitable to evaluate all these catalysts under large A/V conditions too.

As the availability of water molecules at the interface is crucial, flowing electrolyte will refresh the surface with a new electrolyte solution and will lower the chances of additional basification as a consequence of water dissociation which has the tendency to lower the HER kinetics. Hence, the mesoscopic effects of water electrolysers must be taken into account in the evaluation stage too.^[20]^ With research works that are yet to be done and reported in the above‐mentioned directions, it is certain that alkaline water electrolysis (especially the HER part) can be made energy‐efficient in near future.

## 10 Summary and Outlook

Unaffordability of acidic OER electrocatalysts led to the development of alkaline water electrolysers with earth‐abundant OER electrocatalysts albeit that came at a price of compromised HER activity in alkali. Absence of free protons in alkali made the relatively simple and straightforward HER tedious and complex even with the state‐of‐the‐art electrocatalysts. In alkaline HER, to get the necessary localized proton rich environment, it is essential to cause the water dissociation step prior to the classic Volmer step of HER. Hence, in an alkaline medium, the water dissociation and concurrent proton discharge that form hydrogen evolving hydridic intermediates are considered as the Volmer step which controls the overall efficiency of a given electrocatalyst to a greater extent. From recent attempts of using metal hydroxides and their heterostructures as HER electrocatalysts in alkaline media, it is now understood that the metal hydroxide phase facilitates the water dissociation. This is because of their ability to reversibly coordinate with both water and hydroxide ligands which are positioned closer to one another in the spectrochemical series. This has been the reason for all the enhanced HER activity witnessed with Pt‐Ni(OH)_2_ and other related systems. When there is only metal hydroxide, the same metal centres which perform water dissociation do also form hydrogen evolving hydridic intermediates. However, they are relatively stronger in bond strength than the ones formed with a metal atom or the chalcogenide/phosphide anion site in their heterostructures. As a consequence of this, metal hydroxides alone were poor in catalysing HER in alkali. However, if the electronic configuration of the metal ions in the metal hydroxide catalyst being studied is appropriate (i.e. having unpaired *e_g_* electrons to readily donate and dissociate water) and the hydridic intermediate formed with them are of optimal bond strength, they could perform relatively better than other metal hydroxides. An example is Co(OH)_2_ with a conductive carbon support. Having understood that interfacial water structure and its dissociation control the overall efficiency of alkaline HER, we are in an urge to study not only the electrode surface but also the interfacial water structure, electrical double layer, and their reorganization while varying the applied potentials. This can give a more valuable understanding of alkaline HER and the way it does work. This is expected to be accomplished via several in situ and or operando spectroelectrochemical studies to be done in the future. Besides, concentrating on the mesoscopic effect of the electrode and cell is also equally important under large A/V condition to truly realize a better HER performance under industrial operating conditions. With this, we conclude here that alkaline HER will step into a new era of its development in the upcoming years provided that the predicted directions of research in this field will lead so. Eventually, this may end the quest for an energy‐efficient alkaline HER with affordable electrocatalysts (metal hydroxides and their heterostructures) that outperformed Pt/C already at all overpotentials.

## Conflict of interest

The authors declare no conflict of interest.

## Biographical Information

*Sengeni Anantharaj obtained his undergraduate and postgraduate degrees in chemistry from The Presidency College affiliated to University of Madras, Chennai in 2011 and 2013, respectively. Later, he obtained his PhD in 2018 from CSIR‐Central Electrochemical Research Institute (CECRI), Karaikudi, Tamil Nadu, India. Currently, he is availing the prestigious JSPS Postdoctoral Fellowship at Waseda University since January 2019. His research interests include performance‐driven design of electrocatalysts, energy conversion (both fuel‐forming and fuel‐consuming) electrocatalysis, electroactivation, and anodization*.

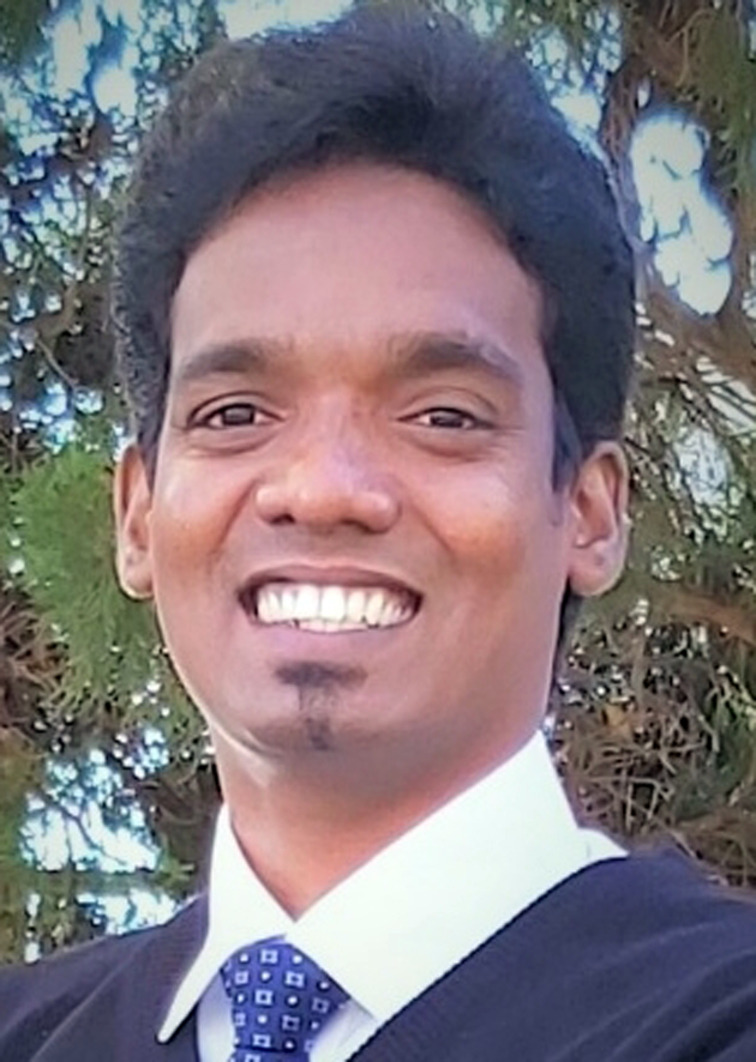

## Biographical Information

*Suguru Noda received his PhD in 1999 from The University of Tokyo, became an assistant professor and associate professor there, and then joined Waseda University in 2012 as a full professor. He is a chemical engineer conducting research in the field of materials processes. He is recently focusing on practical production of carbon and silicon nanomaterials such as carbon nanotubes and silicon films/nanoparticles, and applying these materials to energy and electronic devices*.

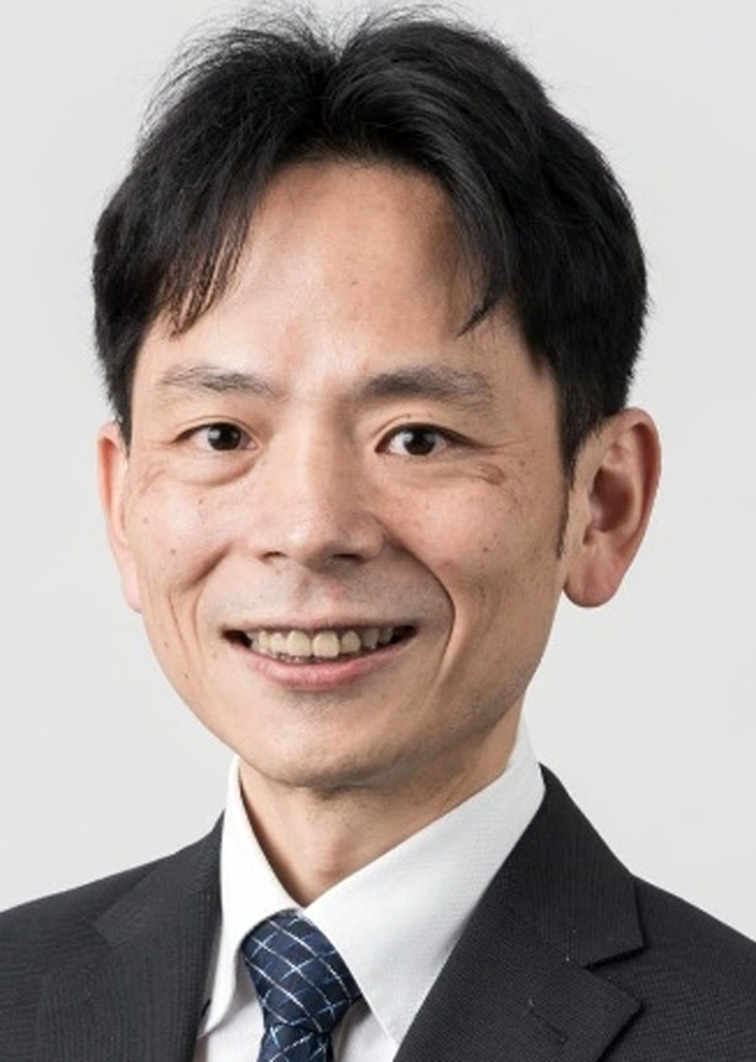

## Biographical Information

*Vasanth Rajendiran Jothi is a Graduate Researcher in the Department of Chemical Engineering at Hanyang University, Seoul. He works in Prof. Yi's group on electrocatalysis projects for hydrogen evolution and water oxidation. He completed his MS at Hanyang University, Seoul, and his undergraduate studies at PSG College of Technology, Kovai, India. He served as a Junior Research Fellow for research projects funded by the Indian Space Research Organization (ISRO). His research interests lie in the electrochemical conversions that are relevant for renewable fuel production*.

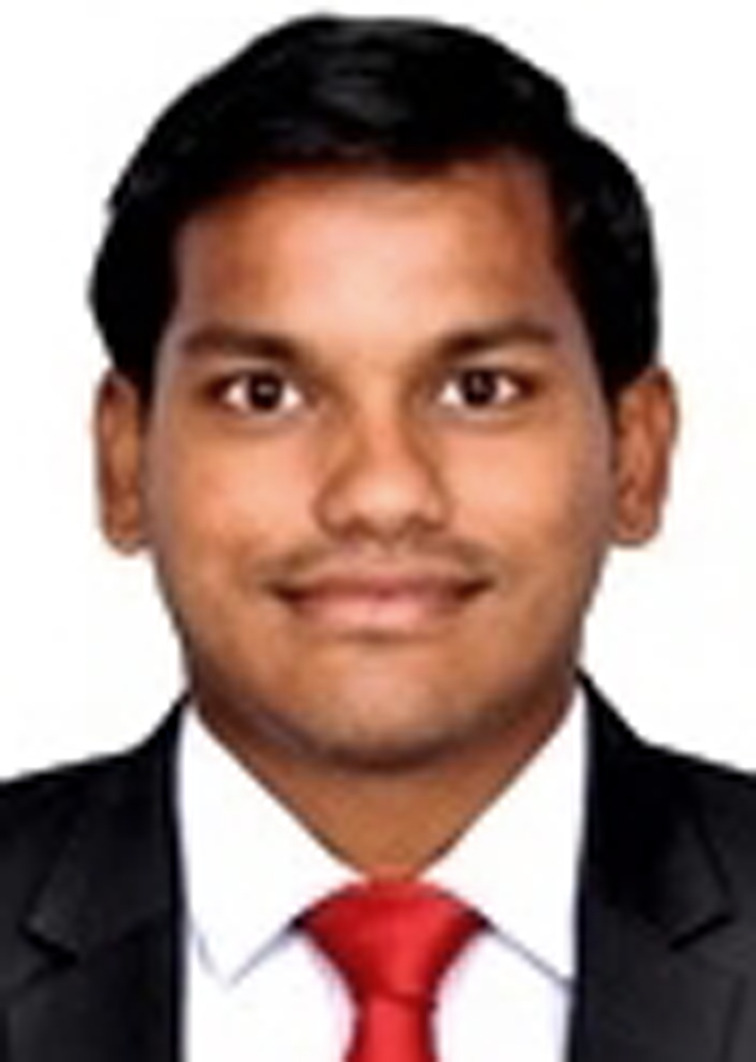

## Biographical Information

*SungChul Yi received his bachelor's diploma in 1981 from the Hanyang University and received his PhD degree (1987) on nonequilibrium thermodynamics and transport phenomena under the mentorship of Prof. R. Rowley at the Brigham Young University. He is currently a Professor of Chemical engineering at the Hanyang University with a research focus on Fuel cells and electrocatalytic water splitting*.

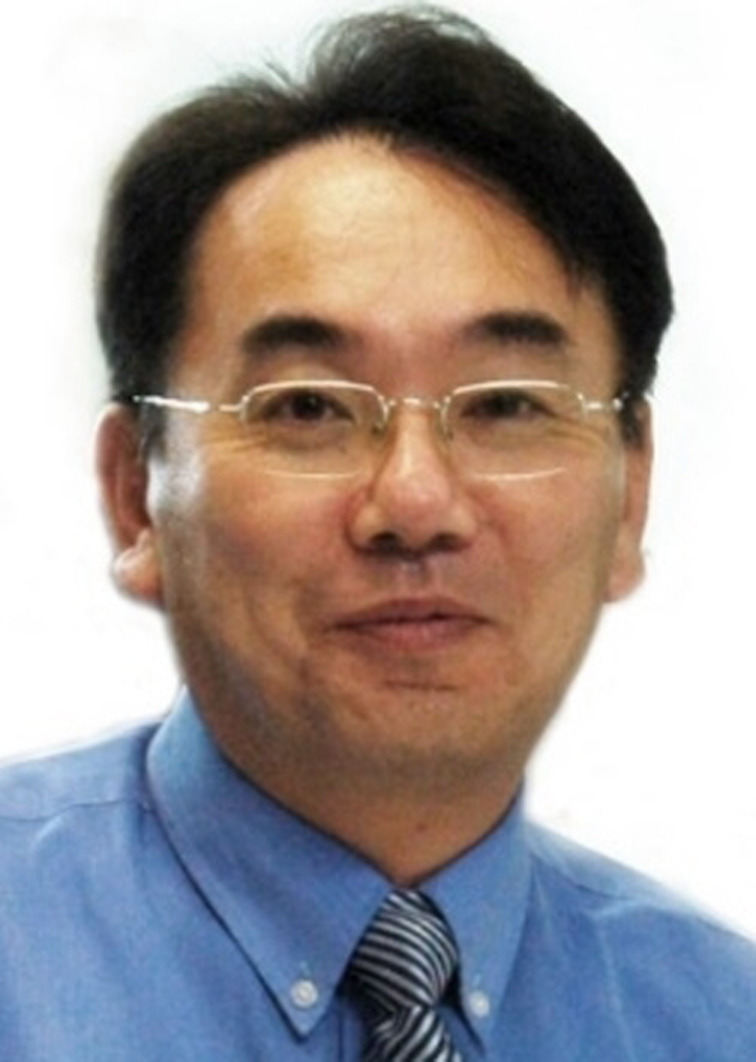

## Biographical Information

*Matthias Driess is a full professor of metalorganics and inorganic materials at the Department of Chemistry of Technische Universität Berlin. He obtained his PhD degree and completed his habilitation at the University of Heidelberg in Germany. He serves as a deputy of the Cluster of Excellence UniSysCat and is a Director of the UniSysCat‐BASF SE joint lab BasCat, and of the Chemical Invention Factory (CIF) for Start‐ups in Green Chemistry*.

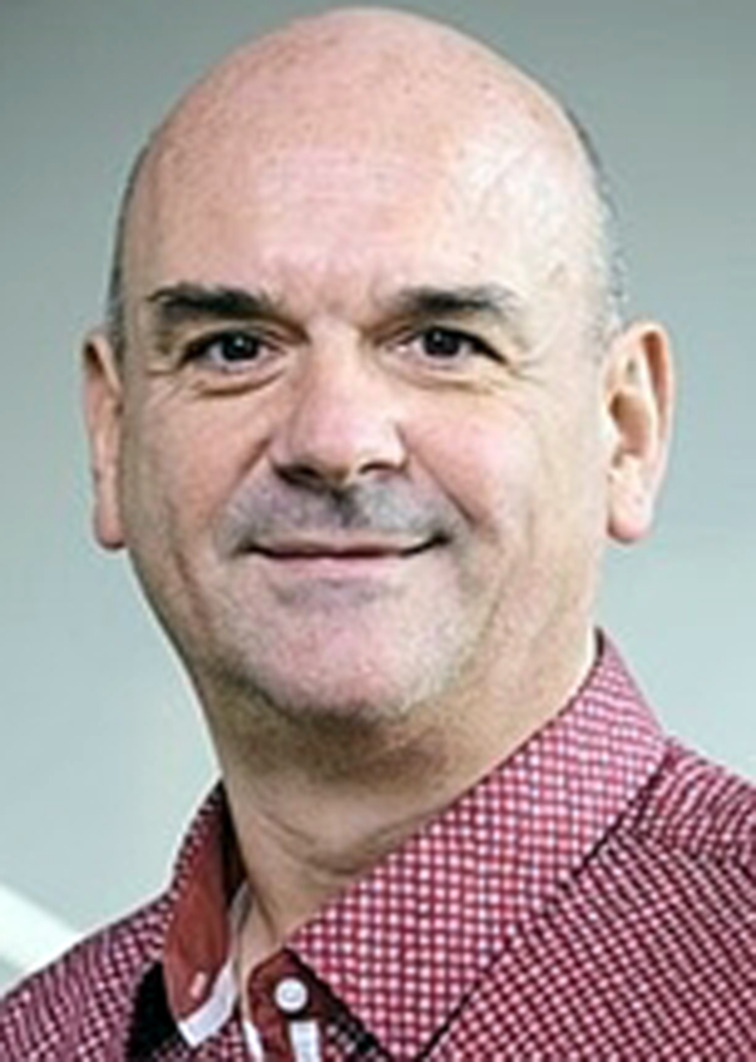

## Biographical Information

*Prashanth W. Menezes received his PhD in 2009 from the Max Planck Institute for Chemical Physics of Solids in Dresden in solid‐state and structural chemistry. He then joined the Technische Universität München in 2010 to work in the direction of inorganic chemistry with a focus on novel materials. In 2012, he joined Prof. Driess at the Technische Universität Berlin and since then serves as a group leader for inorganic materials. His research focuses on the design, development, and structural understanding of novel unconventional catalysts in heterogeneous catalysis, especially in the area of redox oxygen catalysis, and (photo)electrocatalytic water splitting*.